# Multi-omics and synthetic microbial ecology for engineering climate-resilient phytobiomes in cold-arid agroecosystems: current advances and future perspectives

**DOI:** 10.3389/fmicb.2026.1876810

**Published:** 2026-07-02

**Authors:** Vikash Kumar, Faizan Ahmad, Alok Rai, Akhilesh Kushwaha, Kshitij Parmar, Ramji Singh, Ajay Tomar, Chitranjan Kumar

**Affiliations:** 1 School of Horticulture Science and Technology, Kargil Campus University of Ladakh, Kargil, India; 2 Faculty of Agricultural Sciences, GLA University, Mathura, India; 3 Amity Institute of Organic Agriculture, Amity University Uttar Pradesh, Noida, India; 4 Sardar Vallabhbhai Patel University of Agriculture and Technology, Meerut, India

**Keywords:** cold stress adaptation, multi-omics, phytobiome engineering, psychrotrophic microorganisms, synthetic microbial communities (SynComs), system biology

## Abstract

Extreme environmental stressors, including freezing temperatures, strong ultraviolet radiation, and nutrient scarcity, pose a serious threat to global food security in high-altitude cold-arid agroecosystems. Ecological stability depends on the phytobiome, which is made up of plant hosts, their microbiomes, and the edaphic environment. Although plant-associated microbiomes are important in providing stress tolerance, existing management strategies predominantly employ descriptive single-strain inoculants, which often fail under open field conditions due to competitive exclusion and environmental drift. The review summarizes recent mechanistic insights into how psychrotolerant microorganisms modify host physiology to alleviate low-temperature stress. We examine the biophysical and biochemical processes involved, with a particular emphasis on the microbial impact on the host plant’s internal ICE1-CBF-COR transcriptional cascade and redox homeostasis, the role of biofilm-mediated extracellular polymeric substances (EPS) in root-zone thermal buffering, and the kinetic inhibition of ice crystallization by antifreeze proteins. Furthermore, we evaluate how genome-scale metabolic modeling can be combined with sophisticated integrated multi-omics approaches, particularly metagenomics, metatranscriptomics, and metabolomics, to create structurally stable synthetic microbial communities (SynComs), going beyond traditional isolation methods. Lastly, we discuss how regional microbial biobanks and ecological network modeling can maximize consortia persistence, addressing the translational obstacles that prevent laboratory-scale efficacy from reproducing in the field. This synthesis presents a methodical approach for creating robust phytobiomes in vulnerable mountain agroecosystems by moving the emphasis from descriptive cataloging to predictable, function-driven synthetic ecology.

## Graphical Abstract

**Figure:**
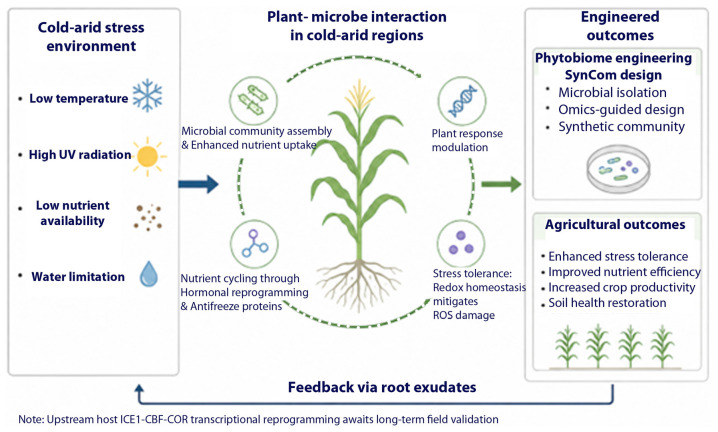
A simplified conceptual overview of plant-microbe interactions and phytobiome engineering techniques in cold-arid agroecosystems: Plant-associated microbial interactions and stress adaption processes are shaped by environmental stressors such as low temperature, excessive ultraviolet (UV) radiation, water scarcity, and low nutrition availability. The overview differentiates between recognised microbial functions and prospective engineering paths, such as SynCom-based methodologies and omics-guided microbial selection, to improve stress tolerance, nutrient-use efficiency, crop production, and soil health. Mechanisms that need more experimental validation are indicated by dashed elements. A framework for designing cold-resilient agroecosystems is based on systems biology. The model combines AI-guided SynCom design with poly-extremophilic microbial diversity from high-altitude habitats. This “Seed-to-Summit” engineering technique ensures global food security in cold-arid locations by circumventing environmental barriers through modulation of the host”s ICE1-CBF-COR signaling and microbial-led metabolic buffering.

## 1 Introduction

The Trans-Himalayan belt and other high-altitude cold-arid agroecosystems are among the most difficult places for terrestrial agriculture. These regions are subject to severe abiotic challenges, including as extreme temperature changes, intense ultraviolet (UV)-B radiation, seasonal moisture deficits, and ongoing macronutrient scarcity. Established crop management paradigms frequently fail in these cold situations because freezing soil temperatures generate major physiological restrictions that hinder essential microbial mineralization and nutrient cycling dynamics (Bhatt et al., 2016). To ensure food production in these vulnerable alpine zones, current agricultural practices must adapt to take advantage of the plant phytobiome, a functional unit composed of the host plant, its related microbial compartments (the rhizosphere, endosphere, and phyllosphere), and the surrounding edaphic environment (Patyal et al., 2025). In this dynamic network, native microbial communities that have adapted to the cold act as essential ecological buffers. These specialist bacteria lessen low-temperature stress by priming the host plant’s natural defenses and altering metabolic pathways, such as increasing the production of antioxidants in the root zone and altering the fluidity of cell membranes. Optimizing these intricate plant-microbe-environment interactions provides a scalable strategy for enhancing crop phenotypic plasticity, stabilizing yield, and re-establishing soil functional ecology in high-elevation desert conditions (Alsharif et al., 2020). Recent research on high-altitude rhizospheres has successfully isolated native microbiomes with specialized molecular toolkits, including antifreeze proteins (AFPs), cold shock proteins (CSPs), and exopolysaccharides (EPS), in addition to conventional plant-growth-promoting traits like indole-3-acetic acid (IAA) and abscisic acid (ABA) production (Chauhan and Pandey, 2024; Rajguru et al., 2024). But merely enumerating these functional traits oversimplifies the serious ecological trade-offs and multi-scale biological constraints that govern microbial engineering in the field. Strong microbial synthesis of IAA, for instance, can simultaneously deplete host carbon reserves in nutrient-deficient alpine soils, increasing plant starvation under high light or cold limitations, and stimulate critical root development to optimize nutrient uptake.

The release of metabolic cryoprotectants like EPS requires a substantial energy investment from the colonizing bacterium. This metabolic outflow often leads to an evolutionary trade-off that significantly reduces the inoculant’s competitive fitness and vegetative growth rate when it is introduced into native soil ecosystems. These energy costs are directly responsible for microbial persistence bottlenecks.

In controlled laboratory or greenhouse settings, individual psychrotolerant strains show significant physiological priming and low-temperature tolerance; yet, their long-term population stability frequently collapses upon open-field inoculation (Kumar et al., 2020). In addition to immediate environmental filtering from different freeze-thaw cycles, introduced inoculants must battle with intense competitive exclusion by well-adapted native microbial consortia. Conflicting empirical evidence highlights this translational gap: field-scale colonization success is rarely consistent with these synthetic expressions, while transcriptionally active, cold-resilient functional genes can be successfully mapped *in vitro* using multi-omics techniques (Varadharajan et al., 2025). Exotic psychrotolerant inoculants result in unexpected ecological drift, affecting the functional structure of the local microbiome for a brief period of time and upsetting native nutrient-cycling cycles. Consequently, to transition from descriptive characteristic summaries to predictive phytobiome engineering, it is crucial to predict how these unique molecular toolkits interact across cellular, community, and field dimensions.

Traditional single-strain bioinoculants must be replaced by multi-omics-validated synthetic microbial communities (SynComs) in order to solve the high failure rates of biological interventions in cold-arid agroecosystems. Traditional single-strain inoculants frequently fail in high-altitude regimes because they lack the functional redundancy and niche adaptation required to withstand extreme environmental conditions, such as rapid freeze-thaw cycles and severe moisture deficits. Furthermore, isolated single strains can be competitively excluded by established, co-adapted native microbiomes. Conversely, rationally created SynComs overcome these limitations by merging keystone taxa that are taxonomically unique but functionally complementary. This multi-strain architecture establishes robust metabolic networks and coordinated biofilm matrices to safeguard the community from random ecological drift and preserve long-term persistence and functional continuity within the host rhizosphere.

A standardized approach must combine high-throughput multi-omics data with computational network modeling to decipher fundamental plant-microbe-environment dynamics to systematically build these robust consortia. This review aims to: (1) identify cold-adapted keystone taxa that regulate phytobiome architecture; (2) elucidate the biochemical pathways underlying low-temperature host resilience; (3) integrate multi-omics datasets (metagenomics, metatranscriptomics, and metabolomics) to map functional microbial traits; and (4) develop a predictable roadmap for custom consortia design. Additionally, we evaluate how these biological frameworks might be integrated with delivery systems for accurate nanobiotechnology. By shielding engineered SynComs from immediate heat shock during inoculation and controlling the release of microbial cells to coincide with the vegetative awakening of high-altitude crops through the use of biodegradable nanogels and protective polymeric matrices, nanobiotechnology overcomes a critical translational bottleneck.

## 2 Review methodology

To find relevant literature exploring the intersections of cold-arid microbial ecology, multi-omics, and synthetic microbial engineering, a semi-systematic literature search was conducted in compliance with the Preferred Reporting Items for Systematic Reviews and Meta-Analyses (PRISMA) 2020 guidelines (Fadiji et al., 2025). Primary records were gathered from four major academic databases: Web of Science, Scopus, PubMed, and Google Scholar (Rajguru et al., 2024). The search was carried out using standardized, formal Boolean search strings that coupled specific cryospheric and agronomic descriptors with multi-omics and synthetic ecology keywords [e.g., (psychrotolerant or cold-arid) and (phytobiome or rhizosphere) and (multi-omics or SynCom)]. The temporal boundary was restricted to a ten-year period (January 2016–April 2026) to prioritize current developments in high-throughput sequencing (Singh, 2021), structural network modeling (Chan et al., 2017), multi-scale multi-omics integration (Geng et al., 2026; Younas et al., 2025), and metabolic reprogramming (Tomar et al., 2025, 2026). The gathered records were subjected to a multi-phase screening process based on exact, predefined eligibility criteria (Abeysinghe et al., 2022). Included were studies that investigated psychrotolerant, psychrophilic, or cold-adapted microorganisms associated with crop plants or high-altitude soil functional ecology using at least one high-throughput omics layer, such as metagenomics, metatranscriptomics, proteomics, or metabolomics (Dhakar and Pandey, 2020; Mukhia et al., 2022; Yadav et al., 2016).

**FIGURE 1:**
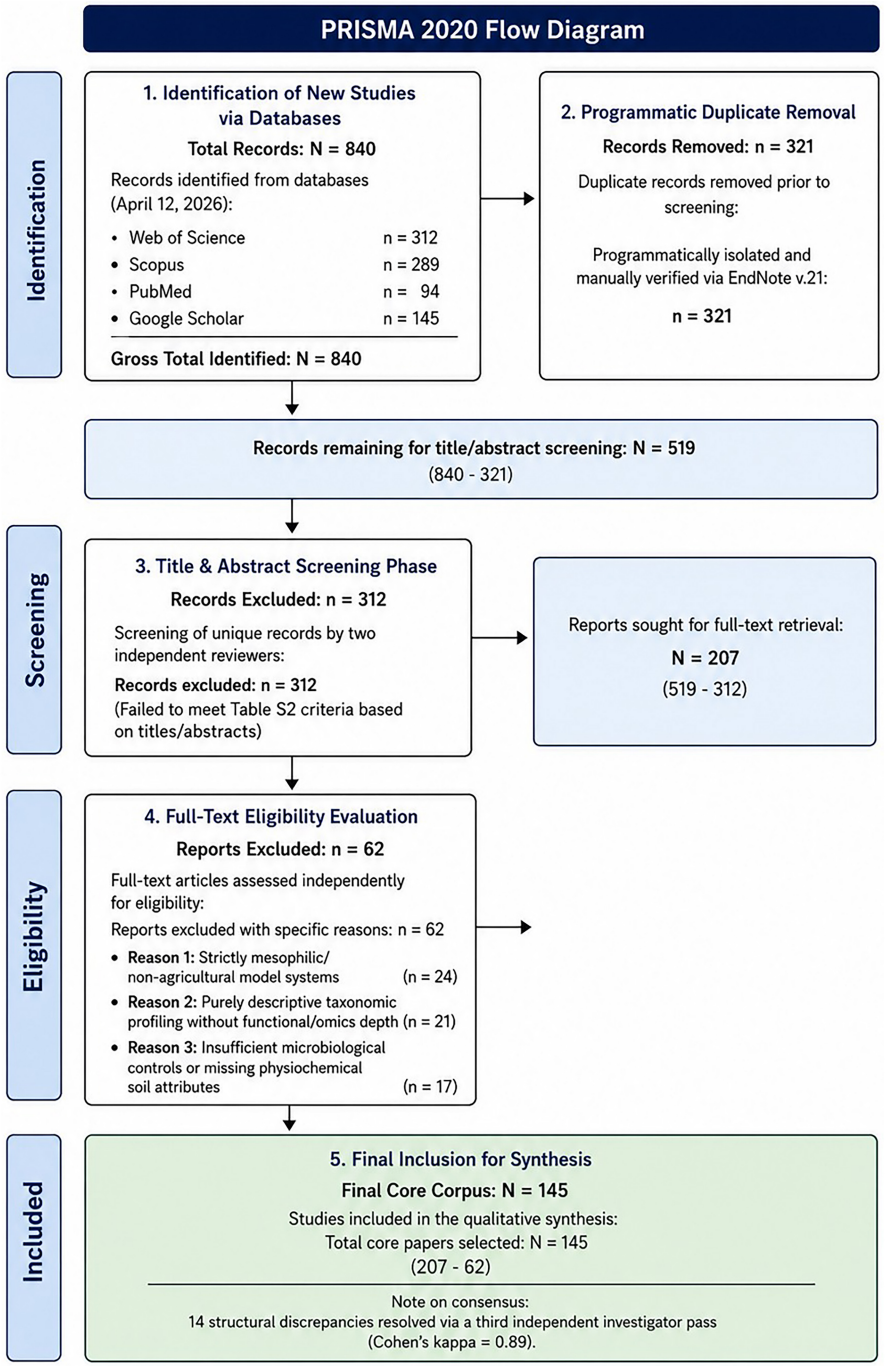
PRISMA 2020 flow diagram illustrating the systematic process for selecting literature. A total of 840 records were found in four databases: Web of Science, Scopus, PubMed, and Google Scholar. After duplicates (*n* = 321) were removed, 519 unique entries were assessed; 312 of these were removed based on a review of the abstract and title. A total of 62 research were excluded based on predefined eligibility criteria after 207 papers received full-text screening. Ultimately, 145 studies were included in the qualitative synthesis. There was a high degree of inter-subject expert agreement (Cohen’s κ = 0.89).

Controlled greenhouse and growth-chamber experiments were deliberately included even though they lacked long-term field validation as long as they offered precise biochemical or physiological mechanistic information about microbial stress mitigation, such as transcriptional modifications or antioxidant defense hierarchies (Jan et al., 2018; Wani et al., 2018; Yang J. et al., 2026). This approach avoids selection against basic physiological literature, which by definition requires controlled conditions (Wendel, 2022). Research was excluded if it contained only non-agricultural mesophilic species, insufficient microbiological controls, or descriptive taxonomic catalogues without functional or metabolic studies (Barros-Rodríguez et al., 2021). Duplicate data were programmatically removed to avoid selection bias. Two reviewers conducted the full-text and secondary title/abstract reviews independently, with a third investigator addressing categorization discrepancies (Fondi et al., 2016). Using this rigorous screening procedure, 145 peer-reviewed original research papers, high-impact reviews, and systems biology case studies were selected for deep qualitative synthesis (Messer et al., 2024). A comprehensive PRISMA screening flow diagram (Figure 1), particular database retrieval yields, indexing dates, and the exact standardized search syntax are all included in Supplementary Tables 1, 2.

## 3 Cold-arid phyto-biomes: functional diversity and architectural composition

### 3.1 Assembly of microbial communities and taxonomic makeup

The establishment of microbial communities is controlled by the convergence of strong abiotic pressures that function as strict environmental filters in high-altitude Tibetan and Trans-Himalayan plateaus, which are archetypal cold-arid agroecosystems (Fadiji et al., 2023; Tuladhar et al., 2023). These fragile alpine soils constitute a classic system dominated by deterministic (niche-based) selection, according to microbial ecology theory, where the active phytobiome is molded more by high physical barriers than by stochastic processes like neutral drift and random dispersal (Ji et al., 2022). The combined stresses of sub-zero temperature regimes, intense UV radiation, persistent moisture deficits, and low organic carbon fractions impose severe ecological filtration (Leung et al., 2020). This deterministic filter excludes mesophilic taxa and only selects psychrotolerant or psychrophilic communities with specific, survival-oriented functional traits (Mukhia et al., 2022; Yadav et al., 2016). After being selected by the first environmental filter, these specialized colonists alter their local environment by creating niches (Afridi et al., 2022a). By secreting highly organized EPS matrices, these early colonists build protective biofilm architectures that physically stabilize soil aggregates, conserve ambient moisture, and shield the community from temperature stress (Bhattacharyya et al., 2023; Costa et al., 2018). This microbial change drives the dynamics of microbiome succession (Bao et al., 2021); the altered microclimate reduces the local environmental stress barrier, allowing more sensitive, non-dominant Actinobacteria and nutrient-transforming taxa to colonize the rhizosphere.

Consequently, through the development of functional redundancy, the community establishes a stable metabolic network capable of metabolic buffering (Santoyo et al., 2021). The idea of ecological resilience during seasonal freeze-thaw cycles is supported by this functional redundancy (Deng et al., 2024). When sudden temperature drops temporarily suppress specific bacterial strains, complementary cold-adapted keystone taxa step in to maintain essential nutrient mineralization and carbon-cycling loops (Kou et al., 2025; Pradhan et al., 2025). It is evident how high-altitude phytobiomes maintain functional continuity in the face of severe environmental stress by concentrating on these deterministic, successional, and network-stabilized assembly dynamics rather than simple taxonomic cataloging.

The phytobiome is an essential evolutionary framework that integrates the host plant’s physiological adaptation with the metabolic flexibility of its microbiome to sustain agricultural yield under severe environmental restrictions (Ullah et al., 2025). In order to strategically tailor these agricultural microbiomes for cold-arid zones, significant ecological knowledge from extreme cryospheric analogues can be directly applied. Glacier ecosystems are typically divided into four geographical zones: supraglacial, englacial, subglacial, and proglacial systems, according to Hu et al. (2022). Highly specialized microbial consortia that have been shaped by distinct, severe environmental stresses can be found in each of these zones. Examining these cryospheric zones as an interconnected system rather than as distinct geological features provides an immediate, translational blueprint for climate-resilient agricultural biotechnology (Hu et al., 2022). Each glacier tier offers a naturally occurring, millions-year-old testing ground for extreme functional features to create SynComs for high-altitude crops. For example, microbial communities with complex photoprotection systems, a hyperactive antioxidant hierarchy, and ice-nucleating or ice-binding proteins in the supraglacial zone are favored by intense solar exposure and repeated freeze-thaw cycles. By utilizing these specific metabolic reactions, crop rhizospheres can be directly shielded from radiative stress and cold shock. Specialized chemolithoautotrophic and heterotrophic communities seen in subglacial and proglacial habitats generate robust nutrient mineralization, phosphorus solubilization, and carbon cycling at temperatures close to freezing. By going beyond simple taxonomic categorization to an integrated, system-wide understanding of these cryospheric zones, agricultural scientists can mine these distinct habitats for specialized psychrotolerant isolates and metabolic processes. These glacier-derived microbial insights offer a reliable toolkit for developing functional phytobiomes that can maintain crop nutrition and metabolic buffering in high-altitude managed farming settings. Instead of being passive residents in these severe environments, microbial communities actively drive biogeochemical cycles and community succession, particularly throughout proglacial and supraglacial zones (Hu et al., 2022). This structural and functional resolution is essential to understanding their roles in maintaining the ecological stability of glaciers and their nearby, downstream terrestrial ecosystems.

By interpreting these intricate cellular networks, researchers can gain a better understanding of how cryospheric pioneer populations produce the basic soil-forming processes that ultimately dictate nutrient availability in high-altitude agricultural soils. Instead of being random, microbial community formation in these regions is regulated by fine-scale niche partitioning. Among the factors that create distinct phyto-biome profiles in high-altitude agricultural environments are altitudinal gradients, edaphic minerals, and specific root exudation patterns. In addition to Ascomycota and Basidiomycota in the mycobiome, important mountain crops like barley, buckwheat, and peas have distinct, localized rhizospheric phytobiomes that are typically dominated by the bacterial phyla Actinobacteria, Proteobacteria, and Firmicutes (Ali and Glick, 2024; Huang et al., 2024; Yadav et al., 2016).

Actinobacteria and psychrotolerant *Pseudomonas* taxa thrive at higher altitudes, according to structural profiling, which clearly shows changes in species abundance along elevational gradients (Yadav et al., 2019). Evaluating these communities requires taking into account significant ecological trade-offs, functional constraints, and structural context dependency. For instance, while high-altitude *Pseudomonas* strains possess vital physiological traits for low-temperature survival, such as membrane lipid desaturation and active DNA repair mechanisms (Yadav et al., 2019), their interactions with the host plant differ substantially. Biochemical and molecular mechanisms of plant-microbe interaction under cold stress have been illustrated in Table 1. Throughout the symbiotic continuum, some *Pseudomonas* genotypes can transform from plant-beneficial mutualists to opportunistic pathogens depending on host immunological signaling, soil nutritional conditions, and temperature fluctuations (La Rosa et al., 2025).

**TABLE 1:** Biochemical and molecular mechanisms of plant–microbe interaction under cold stress.

No.	Mechanism type	Microbial molecules/effectors involved	Affected plant pathways/targets	Functional outcomes	Representative studies (ref. no.)
1	Osmoprotection/osmotic adjustment	Trehalose, proline, glycine-betaine (microbial production or stimulation)	Osmolyte biosynthesis pathways; compatible solute accumulation	Maintains cell turgor, reduces freeze-injury, stabilizes proteins	Jan et al., 2018; Cha-um et al., 2019
2	Exopolysaccharide (EPS) and biofilm formation	EPS, polysaccharide matrices, extracellular polymeric substances	Root surface microenvironment modulation; physical insulation of roots	Micro-thermal buffering, improved soil aggregation, moisture retention	Heredia-Ponce et al., 2021
3	Antifreeze/cold-shock proteins	Antifreeze proteins (AFPs), cold-shock proteins (CspA family)	Inhibit ice crystal growth; stabilize RNA and translation	Reduced intracellular ice damage; better survival during freeze–thaw	Satyakam et al., 2022
4	ROS detoxification/antioxidant augmentation	Catalase (CAT), superoxide dismutase (SOD), peroxidases, glutathione systems	Plant redox signaling networks; upregulation of antioxidant genes	Limits oxidative damage, preserves membrane integrity and photosystems	Qian et al., 2024
5	Hormonal modulation (growth and stress hormones)	IAA, cytokinins, gibberellins; microbial ABA-like compounds; ACC deaminase	Auxin signaling, ABA signaling, ethylene biosynthesis (ACC)	Root growth promotion, controlled stomatal responses, reduced stress ethylene	Roychowdhury et al., 2025
6	ACC-deaminase mediated ethylene lowering	ACC deaminase enzyme (degrades ACC)	Ethylene biosynthesis/signaling (lowers stress ethylene)	Prevents ethylene-induced growth inhibition; sustains root elongation	Shahid et al., 2023
7	Nutrient mobilization (P, N, Fe)	Phosphate-solubilizing enzymes, nitrogenase (N fixation), siderophores	Nutrient-responsive signaling (N, P sensing), iron uptake pathways	Improved nutrient uptake, enhanced chlorophyll synthesis and growth	Yarzábal, 2014
8	Volatile organic compound (VOC) signaling	VOCs (2,3-butanediol, acetoin, terpenoids)	Activation of plant defense and stress-response genes, priming	Systemic tolerance (primed responses), improved stress memory	Montejano-Ramírez et al., 2024
9	Membrane remodeling/lipid desaturation	Induction of unsaturated fatty acid biosynthesis; microbial lipids	Membrane fluidity regulation pathways	Maintains membrane phase behavior at low temperatures; preserves transporters	Wu et al., 2022
10	Chaperones and proteostasis	Microbial secretion of chaperone-inducing signals; microbial chaperones	Upregulation of plant chaperones (HSPs), LEA proteins	Stabilizes proteins, prevents aggregation under cold stress	Storey and Storey, 2023
11	Secondary metabolites and osmoprotective phenolics	Phenolics, flavonoids induced by microbes; microbial antioxidants	Secondary metabolite biosynthetic pathways	UV protection, ROS scavenging, enhanced photoprotection	Wani et al., 2018
12	Ice-nucleation modulation	Antifreeze proteins and ice-nucleation inhibitors	Physical control of extracellular ice formation	Reduced ice nucleation on plant surfaces; safer freezing dynamics	Gharib et al., 2022
13	Mycorrhizal facilitation (P and water)	Hyphal networks, glomalin, hydrophobins	Root hydraulic conductivity, P uptake signaling	Improved water uptake, phosphorus acquisition under cold soils	Khanum et al., 2023
14	Signal priming and systemic induction (MIST)	Microbial elicitors, MAMPs, VOCs	Systemic acquired tolerance-like pathways (defense hormones)	Faster and stronger responses to subsequent cold episodes	Zhao et al., 2020

This table details the biochemical and molecular mechanisms utilized by psychrotrophic and psychrophilic microbes to enhance plant resilience in extreme cold-arid environments. ABA, abscisic acid; ACC, 1-aminocyclopropane-1-carboxylate; AFPs, antifreeze proteins; CAT, catalase; EPS, exopolysaccharides; HSPs, heat shock proteins; IAA, indole-3-acetic acid; LEA, late embryogenesis abundant proteins; MAMPs, microbe-associated molecular patterns; MIST, microbe-induced systemic tolerance; ROS, reactive oxygen species; SOD, superoxide dismutase; VOCs, volatile organic compounds.

Similarly, the presence of powerful, spore-forming taxa like *Bacillus* and *Paenibacillus* highlights a clear evolutionary trade-off (Abeysinghe et al., 2022).

Although endospore-mediated dormancy may allow these organisms to endure extended sub-zero freezing, their metabolic endurance does not ensure successful rhizosphere colonization, competitive niche exclusion, or active plant growth promotion upon spring thaw (Messer et al., 2024). Fast-growing, non-spore-forming psychrophiles frequently outcompete latent spores for root exudates in the early spring, limiting their ability to operate in the rhizosphere (Kou et al., 2025). Future sustainable agroecosystem management relies on cold-tolerant protein engineering (Table 2) and specific formulation chemistry to close the gap between structural discovery and useful agricultural applications (Younas et al., 2025). To effectively manage and implement functional synthetic consortia in cold-adaptive zones, targeted inoculation procedures that take these colonization bottlenecks and ecological limitations into consideration must be developed. In delicate high-altitude regions with moisture deficiencies, microbially mediated soil structural engineering is essential (Prakash et al., 2026) for preserving hydraulic integrity. By secreting compounds that stabilize macroaggregates and minimize water loss, biofilm-producing bacteria and arbuscular mycorrhizal fungi improve soil hydrology. For synthetic consortia to be structurally stable and climate-resilient, these hydro-microbial feedbacks must be integrated along ecological gradients.

**TABLE 2:** Comparative synthesis of phytobiome engineering strategies in cold-arid agroecosystems.

S. no.	Engineering approach and scope	Targeted microbial taxa	Core mechanisms of cold and arid adaptation	Associated omics evidence	Representative crop systems	Validation status	Translational readiness and limitations	References
1	Single-strain bioinoculants (PGPR) Localized rhizosphere/phyllosphere targeting via traditional delivery modes.	Psychrotrophic strains of *Bacillus*, *Pseudomonas*, *Arthrobacter*, and *Exiguobacterium*.	Production of ACC-deaminase, IAA, cold-active osmolytes (proline, trehalose), and scavenging of ROS via antioxidant enzymes.	Transcriptomic profiling of *Aegilops-Triticum* composites; identification of bacterial *acdS* and *iaaM* gene expressions under chilling stress.	Wheat (*Triticum aestivum*), Barley (*Hordeum vulgare*), Tomato (*Solanum lycopersicum*).	Extensive field, greenhouse, and seed-treatment validation globally.	High (TRL 7–8) Limitations: poor persistence under extreme field diurnal temperature fluctuations; high native microbiota competition.	Yadav et al., 2016; Rondón et al., 2019; Gulati et al., 2024; Vaghela et al., 2026
2	Multi-strain functionally complementary consortia Co-inoculation of metabolic guilds to provide functional redundancy.	Consortia containing *Bacillus* spp., *Pseudomonas* spp., and cold-tolerant diazotrophs.	Synergistic phosphate solubilization, multi-pathway induced systemic resistance (ISR), and synthesis of diverse structural exopolysaccharides (EPS).	Metagenomic profiling of arid soils; metaproteomics validating the concurrent up-regulation of nitrogenase and mineral-solubilizing pathways.	Alfalfa (*Medicago sativa*), Legume systems, and cold-desert horticultural crops.	Mixed validation: robust greenhouse synergy, variable field reproducibility.	Medium-High (TRL 6–7) Limitations: in-storage strain incompatibility; complex quality control/standardization; regulatory approval hurdles.	Santoyo et al., 2021; Gurubasajar and Basaiah, 2025; Parveen et al., 2026
3	Endophyte enrichment and core colonizers Inoculation of systemic niche occupants immune to immediate soil fluxes.	Endophytic *Arnebia euchroma* isolates, *Pseudomonas* endophytes, and specialized fungal endophytes.	Direct modulation of host calcium signaling networks (Ca∧{2 + }-dependent protein kinases), up-regulation of host ICE-CBF-COR antifreeze cascades, and internal membrane protection.	Host-endophyte co-transcriptomics and comparative proteomics revealing up-regulated chloroplast protection proteins and MAPK cascades.	Medicinal cold-desert flora (*Arnebia*), Alpine cereals, and Raspberry/ Blackberry crops.	Primarily laboratory, tissue-culture, and controlled nursery-stage validations.	Medium (TRL 5–6) Limitations: high genotype-specific colonization variability; restricted host range; biosafety clearance for internal tissue colonizers.	Jain et al., 2021; Chaudhary et al., 2022; Wang et al., 2026; Handoko and Lin, 2025
4	Arbuscular mycorrhizal fungi (AMF) networks Glomales-mediated macro-matrix engineering for moisture and nutrient pathways.	*Rhizophagus irregularis*, *Funneliformis mosseae*, and indigenous cold-adapted glomales.	Hyphal extraction of recalcitrant phosphorus; glomalin-driven soil macro-aggregation; host membrane stability through lipid composition remodeling.	Genome-scale metabolic modeling of mycorrhizal transport; transcriptomic mapping of host abscisic acid (ABA) and aquaporin gene shifts.	Maize (*Zea mays*), high-altitude vegetables, and perennial forage crops.	Well-validated in micro-plots and alpine pot trials; low large-scale field data in true cryic zones.	Medium (TRL 6) Limitations: extremely slow spore germination/hyphal extension in sub-zero soils; high cost of axenic mass multiplication.	Khanum et al., 2023; Ding et al., 2025; Nie et al., 2024; Makris et al., 2026
5	Advanced cryoprotective formulations and encapsulation Polymeric matrix immobilization to cushion thermal shock during storage/release.	High-performance PGPR strains wrapped in alginate, chitosan, or biopolymer blends.	Physical shielding against rapid freeze-thaw cycles; controlled release kinetics synchronized with root exudation phase-changes.	Viability-qPCR and LC/MS-based metabolomic fingerprinting tracking cellular stress memory and membrane structural integrity during desiccation.	Broad-spectrum applicability (seed coating, granules for dry-land cereals).	Laboratory scale-up completed; niche translational field validation underway.	Medium-High (TRL 6–7) Limitations: high initial material and industrial formulation scale-up costs; precision timing of degradation kinetics in cold soils.	Hu et al., 2023; Colin et al., 2025; Liu et al., 2025
6	Omics-Designed Synthetic Communities (SynComs) *De novo* construction of minimal, stable, functional microbial networks.	Rationally selected species crossing *Bacteroidetes*, *Firmicutes*, and *Proteobacteria* phyla.	Multi-tier ecological niche saturation; metabolic cross-feeding maximizing network stability against structural freeze-thaw cycles.	Metatranscriptomics and predictive metabolic flux models (*FBA*) validating stable community co-occurrence and carbohydrate processing networks.	Model crops initially (*Arabidopsis*), transitioning to high-altitude protected leafy vegetables.	Dominated by highly controlled laboratory microcosms and gnotobiotic systems.	Low-medium (TRL 4–5) Limitations: massive translational gap from sterile lab media to open, highly unpredictable natural cold soils.	Gibbons and Gilbert, 2015; Contreras-Salgado et al., 2024; Parveen et al., 2026
7	Targeted microbiome editing and trait transfer Direct genetic/epigenetic modification of key phyto-biome modules (GM & Non-GM).	Standard model psychrotrophs (*Pseudomonas* spp., *Bacillus subtilis*).	Over-expression of specific antifreeze proteins (AFPs), hyper-accumulation of cell-surface extracellular polymeric substances (EPS).	CRISPR-Cas9 mapping, targeted metagenomics tracking plasmid persistence, and horizontal gene transfer (HGT) kinetics in cold profiles.	High-value cash crops and high-altitude glasshouse crops.	Strictly confined to containment facility laboratory experiments and biosecure advanced trials.	Low (TRL 3–4) Limitations: severe ethical, biosecurity, and regulatory blockades; high ecological risk of transgene escape into pristine alpine biomes.	Gharib et al., 2022; Satyakam et al., 2022; Madushani et al., 2026
8	AI/ML-guided predictive strain selection Computational pipeline deployment to bypass empirical screening loops.	Virtual data models managing vast libraries of psychrotolerant isolates.	*In silico* matching of soil physical parameters (pH, organic carbon, frost-free days) with microbial genomic functional traits.	Deep learning architectures utilizing multi-omics inputs (metagenomics + metadata) to accurately predict plant disease resistance and stress survival indices.	Macro-regional predictive models for wheat, legumes, and brassica production systems.	Validated via computational cross-validation and retrospective match-testing with legacy field datasets.	Medium (TRL 5) Limitations: constrained by the acute shortage of high-resolution, long-term multi-omics datasets from fragile cold deserts.	Mohamedikbal et al., 2025; Thingujam et al., 2025; Kumar et al., 2026

This table provides a comparative analysis of diverse phyto-biome engineering strategies, ranging from traditional bioinoculants to advanced AI-driven synthetic approaches, tailored for cold-arid agroecosystems. ACC, 1-aminocyclopropane-1-carboxylate; AFP, antifreeze protein; AI/ML, artificial intelligence/machine learning; AMF, arbuscular mycorrhizal fungi; EPS, extracellular polymeric substances; FBA, flux balance analysis; GM, genetically modified; IAA, indole-3-acetic acid; ISR, induced systemic resistance; MAPK, mitogen-activated protein kinase; PGPR, plant growth-promoting rhizobacteria; ROS, reactive oxygen species; TRL, technology readiness level.

The metabolic architecture of cold-arid soils is reinforced by the archaeal phylum Thaumarchaeota in addition to bacteria and fungi. These ammonia-oxidizing archaea assist low-temperature nitrification, an essential mechanism that maintains nitrogen flow in nutrient-lean environments where bacterial mineralization is often prevented (Vega-Celedón et al., 2021; Crivelli et al., 2024). When considered collectively, this taxonomic diversity builds a robust functional network that provides the essential metabolic buffering that plants in the world’s most vulnerable agricultural frontiers require to thrive.

### 3.2 The mechanistic underpinnings of cold resilience and functional features

Rather than being a collection of distinct microbial characteristics, cold-arid phytobiome resilience is a tightly coordinated, multi-scale metabolic response intended to maintain cellular integrity under extreme temperature instability (Ullah et al., 2025). To preserve functional continuity, psychrotolerant PGPR that are indigenous to high-altitude Himalayan regions (Bhattacharyya et al., 2023), use a highly synchronized dual-action survival strategy that combines extracellular niche engineering with biophysical membrane stabilization (Yadav et al., 2019).

#### 3.2.1 Biophysical kinetics of membrane homeostasis and cryoprotection

Cold-tolerant strains quickly alter their cellular architecture and protein expression kinetics to resist abrupt temperature reductions and avoid structural collapse (Ji et al., 2022). Specialized CSPs function as RNA chaperones, attaching to specific mRNA transcripts to destabilize secondary hairpin structures in order to maintain continuous translation efficiency under low-temperature stress (Yang J. et al., 2026). Simultaneously, the hydrophobic flat facets of secretome-localized AFPs bind directly to newly produced ice crystal nuclei, lowering the ice recrystallization temperature and non-colligatively inhibiting ice crystal formation (Dhakar and Pandey, 2020). This kinetic inhibition preserves the integrity of cell walls and prevents mechanical shearing of root tissues (Jan et al., 2018). Psychrotolerant strains upregulate desaturase enzymes (Δ^9^- and Δ^12^-desaturases) at the membrane level to convert fatty acids from a stiff, gel-like state to a fluid, liquid-crystalline one by adding cis-double bonds to acyl chains (Yadav et al., 2019).

The intracellular build-up of suitable solutes, mainly proline and trehalose, complements this homeoviscous adaptation (Zhang et al., 2023). By replacing water molecules surrounding protein structures, these osmoprotectants create essential hydrogen bonds that preserve appropriate protein folding kinetics and safeguard cellular osmotic equilibrium during freezing-induced desiccation (Wani et al., 2018). Psychrotolerant bacteria carry out a highly coordinated, multi-pathway survival response to freeze-thaw shock. In order to preserve a fluid, liquid-crystalline condition and avoid hard membrane gelation, the cell first initiates desaturase activation, which introduces cis-double bonds into fatty acid acyl chains. In order to offer mechanical shear suppression against root tissue damage, secreted AFPs bind directly to the flat facets of developing ice nuclei, preventing ice recrystallization. Lastly, during freezing-induced desiccation, the intracellular build-up of suitable solutes such as proline and trehalose induces hydration shell substitution, creating critical hydrogen bonds surrounding proteins to maintain cellular osmotic balance and important folding kinetics.

#### 3.2.2 Kinetic preservation

To convert molecular discoveries into reliable field-scale agricultural interventions, a shift from descriptive taxonomic profiling to a predictive, systems biology paradigm (Fondi et al., 2016) driven by multi-scale meta-omics integration is required (Geng et al., 2026). High-resolution multi-omics has already demonstrated how specific modifications in intracellular pathways lead to macroscopic performance reductions with seasonal cooling in parallel engineering systems (Messer et al., 2024). For example, studies show that certain cold-adapted keystone strains maintain baseline functional stability at 4 °C by selectively upregulating pathways that regulate the generation of cryoprotectants, antioxidant defenses, and membrane fluidity (Messer et al., 2024). Importantly, because these responses are very strain-specific and context-dependent, transcriptome activation may not always translate into ecosystem-level resilience.

The high energetic cost of low-temperature survival routes can significantly decrease overall agronomic and ecological performance, forcing microbial communities to make challenging bioenergetic trade-offs when resources or nutrients are few. This multi-scale microbiome engineering strategy allows researchers to establish distinct metabolic signaling thresholds in high-altitude agroecosystems (Younas et al., 2025). Although high-throughput genomic and transcriptomic sequencing datasets have extensively documented individual cold-responsive components, the primary challenge is integrating these various data layers using genome-scale metabolic models and flux balance analysis (FBA) (Fondi et al., 2016). Treating the microbial cell as an integrated, synchronized thermodynamic unit allows for the precise prediction of metabolic network reactions to different heat regimes (Fondi et al., 2016). This structural modeling approach provides the precise theoretical foundation (Messer et al., 2024) required to bridge the gap between fundamental molecular research and the synthetic production of resilient, climate-resilient microbial consortia.

#### 3.2.3 The theory of biofilm-buffer

By secreting EPS, PGPR alters both internal metabolic pathways and the physical structure of the rhizosphere. These polymers provide functional robustness via three distinct biophysical mechanisms. First, extremely concentrated EPS matrices within localized micro-domains may produce a little freezing point depression of the surrounding root-zone border layer by interfering with initial ice crystal nucleation networks (Costa et al., 2018). However, local EPS concentration gradients, ambient soil moisture retention, and matrix-specific physicochemical interactions all significantly influence the size of this thermal depression *in situ*. Second, the development of dense bacterial biofilms creates a micro-thermal niche, which stabilizes the immediate root-soil interface and reduces the severity of daily temperature fluctuations (Bhattacharyya et al., 2023). Third, EPS secretion maintains essential hydraulic connectivity within vulnerable topsoils by stabilizing soil aggregates and preserving pore-network continuity. This matrix-mediated structure ensures that localized moisture remains accessible to the root system in order to withstand the desiccating effects and structural instabilities caused by frost-heaving cycles.

### 3.3 The endophytic interface: integration of multi-trophic functions

Within the phytobiome, the endophytic compartment of the plant-associated microbiome serves as a highly specialized functional interface. By establishing an intracellular niche that partially protects the microbial partner from unstable, adverse exterior macro-environments, endophytes internalize adaptive advantages directly within host tissues (Panwar et al., 2025). Converting these endophyte-mediated benefits into dependable field-scale solutions is still a major challenge, though. Successful inoculation is never always certain since host genotypic compatibility, temperature variations, localized soil chemistry, and intense ecological competition with well-adapted native microbiomes all closely regulate stable endophytic colonization and seasonal persistence (Kumar et al., 2025).

#### 3.3.1 High-altitude taxa and the limits of genomic convergence

Certain species, such as *Pseudomonas, Arthrobacter*, and *Microbacterium*, often dominate the endophytic profiles of robust medicinal plants (Jain et al., 2021) like *Arnebia euchroma* in specialized high-altitude settings, such as the cold desert zones of Ladakh. These consortia clearly show functional convergence rather than being stochastic assemblages, with limited core genomes adapted for specialized nutrient uptake and phytohormone modulation under heat stress. For instance, these taxa produce cytokinins and IAA to regulate root architecture while also using high-affinity siderophores to assist store iron in alkaline, cold-locked soils (Yadav et al., 2016). The practical application of these strains is severely restricted by host specificity; a keystone endophyte that demonstrates strong multi-scale persistence and growth-promoting efficacy in its native alpine host often fails to colonize non-cognate crop species or persist across distinct seasonal turnovers.

#### 3.3.2 Oxidative defense and redox regulation

The compounding effect of low-temperature stress and intense sun radiation is a crucial, high-impact gap in the existing rhizosphere ecology. High-altitude endophytes frequently alter the host’s antioxidant network, therefore it’s important to evaluate the relationship between microbial inoculation and enzyme activity (such SOD, CAT, and APX) carefully. Rather than usually indicating greater tolerance, increased antioxidant enzyme activity may just indicate an advanced stage of oxidative stress exposure and intracellular macromolecular damage (Berrios and Rentsch, 2022). To completely distinguish adaptation from cellular harm, it is crucial to look at both the absolute overexpression of host enzymes and the net metabolic effects. Instead of merely producing an unchecked increase in reactive oxygen species (ROS) generation, symbiotic endophytes lower these parallel demands by proactively priming the host’s mitogen-activated protein kinase (MAPK) signaling pathways and metabolic cascades prior to severe climate shifts (Jalmi and Sinha, 2015). This targeted bacterial manipulation maximizes the kinetic scavenging of superoxide radicals and hydrogen peroxide (H_2_O_2_) generated during simultaneous cooling and UV-B exposure (Jain et al., 2021; Wani et al., 2018). Ultimately, high-altitude endophytes prevent the systemic oxidative collapse frequently observed in uninoculated, climate-stressed hosts by establishing a controlled, homeostatic balance between ROS production and scavenging capacity (González et al., 2024).

#### 3.3.3 The multi-trophic continuum

The density of arbuscular mycorrhizal fungi (AMF) drops at very low temperatures; however, they play a significant function as hydraulic bridges. In frozen soils, where water is physically available but not biologically accessible, AMF improves phosphate absorption and root hydraulic conductivity (Nie et al., 2024), offering a crucial drought-resistance mechanism. These interactions provide a predictive engineering paradigm that integrates the physiological decision-tree of the plant with microbial functional activities such as osmoprotection, hormone modulation, and redox management (Camaille et al., 2021). To create translational strategies in fragile alpine ecosystems, we must go beyond simply listing species and actively predict functional results.

### 3.4 Biochemical and molecular adaptations: the mechanistic synergy

Microbial adaptation to cold-arid stresses is a highly integrated, whole-cell metabolic reconfiguration rather than a collection of discrete survival features. The core of this defense is homeoviscous membrane adaptation, which maintains membrane fluidic integrity and prevents the structural rigidity caused by heat dips. An elevated ratio of unsaturated fatty acids makes it feasible. This lipid stabilization collaborates with the intracellular build-up of compatible solutes such as glycine betaine and trehalose, which function as multi-modal thermodynamic stabilizers, to preserve native protein folding kinetics and nucleic acid structures under severe desiccation stress (Cha-um et al., 2019; Mehta and Vyas, 2023). Rather than operating as distinct systems, these intracellular defenses are physically anchored by the previously mentioned extracellular matrix (AFPs and EPS) and redox-scavenging networks (SOD and CAT). The real-time regulation of these pathways creates an integrated molecular connection between the endophyte and the host. This connection is immediately reflected in the host plant’s secondary metabolome; some signaling volatile organic compounds and secondary metabolites generated by endophytic Actinobacteria and *Pseudomonas* strains serve as inter-kingdom elicitors (Kanjana et al., 2026; Yang Y. et al., 2026). By actively upregulating the host’s endogenous osmoprotective pathways and altering hormonal networks, these microbial compounds display a co-evolved metabolic continuity that shields the entire holobiont against high-altitude thermal stress.

### 3.5 Microbial interactions: from cataloging to predictive systems biology

The transition from individualized competitiveness to system-level modularity is an essential paradigm for understanding life in high-altitude environments. Ecological network research has shown that cold-arid soil communities frequently form small-world networks with densely interconnected modular units (Fadiji et al., 2025). Under the influence of high-affinity quorum sensing mechanisms, some keystone taxa—usually non-dominant Actinobacteria or specialized Proteobacteria—that are assumed to serve as metabolic hubs within these modules may contribute to community stabilization and nutrient transformations (Bao et al., 2021). Deterministic selection pressures, specifically, different freeze-thaw cycles and severe matric water potential, have a significant impact on these community structures by favoring specific functional traits over taxonomic variety (Kou et al., 2025). In reaction to low-temperature stress, plants actively alter their root exudate profiles by secreting more specific sugars and organic acids. Psychrophilic populations may be drawn to this confined, bidirectional signaling nexus (Yang et al., 2025). This synchronization between the metabolic cycles of the phytobiome and physical soil transitions is a compelling new notion that implies a developed, predictive adaptive response rather than only a passive physiological reaction.

### 3.6 Global convergence and the minimal functional genome

Comparative meta-analyses of the Tibetan Plateau, Antarctic Dry Valleys, and Arctic tundra show some functional convergence among cold-adapted microbiomes despite their disparate geographic and evolutionary origins (Ji et al., 2022). However, recent studies suggest that rather than a strictly genetic design or a universally conserved minimal functional genome, cold tolerance depends on context-specific, highly redundant, strain-dependent processes. Because of intermittent human inputs, high-altitude agroecosystems maintain higher taxonomic variation and functional redundancy than virgin polar deserts (Barros-Rodríguez et al., 2021). Similarities can be observed in high-latitude marine habitats, where polar bacteria and microalgae exhibit significant molecular plasticity, characterized by modifications in lipid metabolism and the overexpression of defensive systems such as antioxidant enzymes, ice-binding proteins, and molecular chaperones (Lauritano et al., 2020). Although particular gene gains and losses linked to these cold-adapted features have been found using omics approaches, a significant percentage of environmental sequences are still functionally unknown (Lauritano et al., 2020). Despite these developments, the absence of a predictive systems biology network that connects metagenomic potential with real-time *in situ* phenotypic reality remains a crucial conceptual gap in our understanding of inter-community dynamics under varying environmental stressors. To overcome the drawbacks of cultivation-dependent validation, future high-impact research must move toward machine learning (ML)-assisted multi-omics integration (Ejaz et al., 2024; Parveen et al., 2026). This computational framework is more than just a catchphrase; it necessitates feeding intricate predictive algorithms with high-resolution multi-omics training datasets, particularly paired seasonal freeze-thaw soil metagenomes and host plant metatranscriptomics (Deng et al., 2024; Thingujam et al., 2025). Both biological traits (such as expression levels of ACC-deaminase, CSP/AFP gene cassettes, and abundance patterns of psychrotrophic keystone operational taxonomy units) and edaphic constraints (such as matric water potential, soil organic carbon, and diurnal thermal flux rates) (Younas et al., 2025). These multi-dimensional data arrays can be modeled using constraint-based optimization and flux balance frameworks, like SteadyCom (Chan et al., 2017), to mathematically simulate microbial community abundances while ensuring thermodynamic and structural stability under thermal shock (Passi et al., 2021).

The application of these ML-driven models has significant computational and translational constraints. One significant disadvantage is the high proportion of dark matter in cold-desert metagenomes, sequences that are still functionally unknown and cannot yet be connected to recognized metabolic pathways (Lauritano et al., 2020). Model training is severely hampered by the absence of uniform field-scale validation plots and variations in data quality across sequencing methods (Smith et al., 2026). These algorithmic limitations must be solved by merging targeted field-scale experiments with predictive ML outputs in the next generation of region-specific, context-aware bioformulations tailored for delicate alpine agroecosystems.

## 4 Mechanisms of plant-microbe interaction under cold stress

Under severe heat deficiencies, a highly coordinated, multi-tiered stress-buffering network mediates the symbiotic interaction between plants and the psychrotropic bacteria they are associated with. This physiological barrier specifically reduces the three primary physical dangers associated with cold stress: fatal ice nucleation, plasma membrane rigidification, and cellular dehydration. Instead of acting as discrete inputs, microbial metabolites act as external signaling agents that systematically alter the host’s systemic transcriptional landscape. Microbial release of specialized AFPs and EPS is an essential cellular defense mechanism (Munir et al., 2022). These biopolymers physically attach to growing ice crystals to obstruct their growth and recrystallization processes, preventing the disastrous mechanical rupture of plant cell walls and membranes (Shaffique et al., 2022).

**FIGURE 2:**
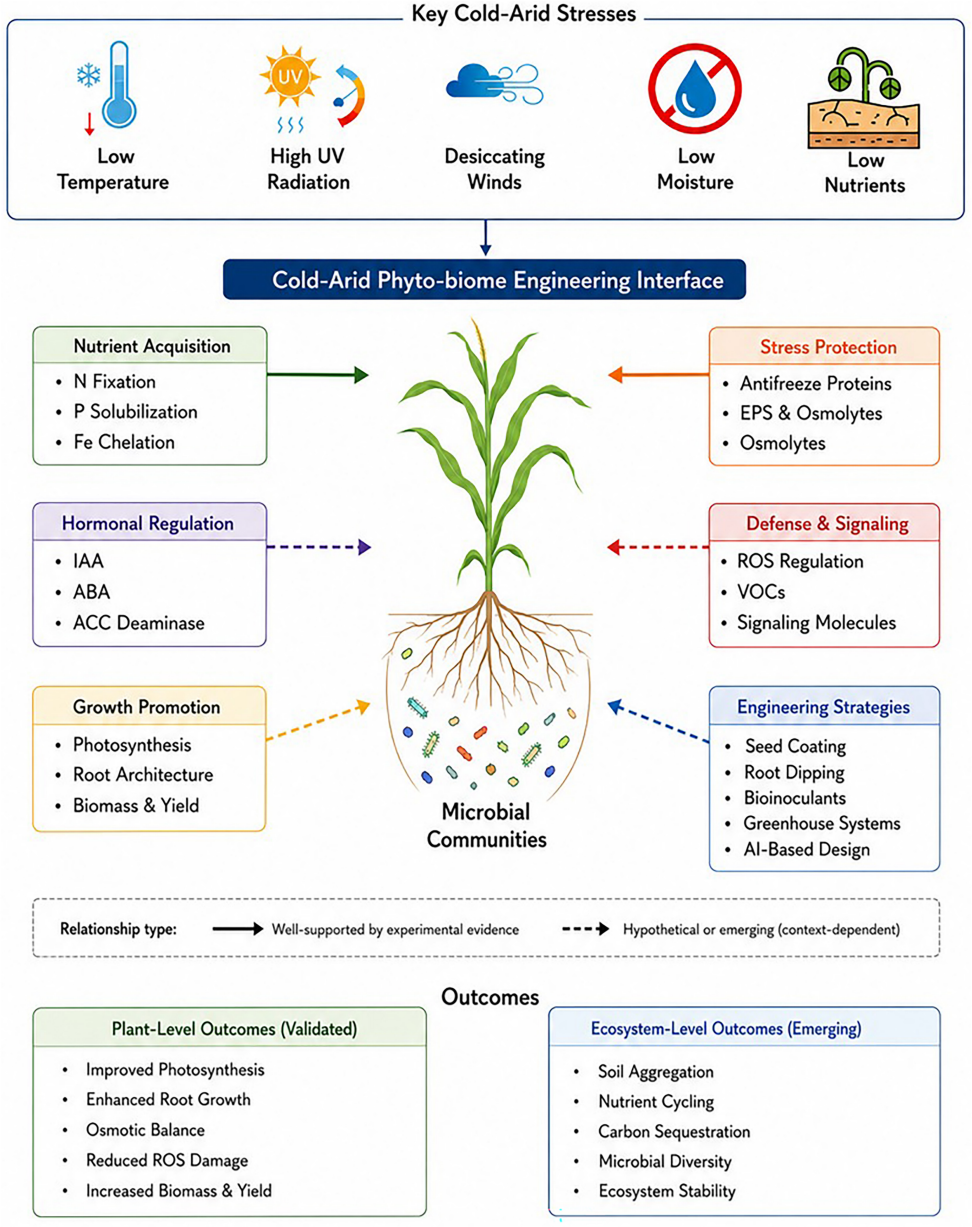
Conceptual framework showing the interaction of the phytobiome of cold-arid, and high-altitude agroecosystems. Numerous abiotic challenges, such as low temperatures, intense UV radiation, desiccating winds, limited moisture availability, and nutrient-deficient soils, are experienced by plants in these settings. The plant host, along with the rhizosphere, endosphere, phyllosphere, and surrounding abiotic environment, make up the phytobiome, an interconnected and dynamic ecological system. Through a variety of plant-microbe interactions, microbial populations support defense signaling, hormone regulation, stress tolerance, and nutrient uptake. Together, these mechanisms promote plant development, physiological adaptability, and ecological stability in cold-arid environments. While recognizing that some interactions are still context-dependent and need more experimental validation, the schematic provides a conceptual overview of prospective phytobiome engineering strategies for creating more adaptive and sustainable cold-arid agricultural systems.

Concurrently, the microbially generated accumulation of appropriate osmolyte solutes, such as trehalose, proline, and glycine betaine, stabilizes the host’s plant lipid bilayer and maintains cellular turgor under stress. This metabolic synergy directly affects the host’s macromolecular defense. Microbial inoculation is a potent biochemical primer that accelerates the host’s natural synthesis of osmoprotectants, including polyols and soluble sugars (Mehta and Vyas, 2023). By decreasing the cytoplasmic freezing point and safeguarding intracellular machinery, this symbiotic relationship effectively transforms a passive, energy-draining survival response into an active, microbially boosted defensive mechanism. Beyond these cellular limits, EPS-mediated bacterial biofilms dramatically change the immediate rhizosphere’s physical structure. Instead of serving as inert covers, these structured matrices provide dynamic micro-habitats that modify the biophysical properties of the surrounding soil through enhanced hydraulic continuity and localized heat buffering. Thermal buffering reduces abrupt, daily changes in soil temperature by forming a micro-scale insulating layer inside the biofilm matrix. Hydraulic continuity concurrently improves macro-soil aggregation and moisture retention while reducing cold-induced desiccation and maintaining a constant water potential gradient across the critical root-soil interface (Lee and Cho, 2025). The integrated routes of this rhizospheric and intracellular protective network, along with their intended physiological effects, are shown in depth and visually in Figure 2 and Table 1 (Ullah et al., 2025).

Microbially driven changes in the host’s phytohormonal network significantly influence plant growth and structural development under low-temperature deficits. However, these hormone-driven pathways are very concentration-dependent and context-specific, frequently requiring a precise physiological trade-off between growth promotion and stress survival. The microbial release of auxins (or IAA), cytokinins, and gibberellins can function as external developmental signals to increase root architecture and enhance nutrient uptake in extremely viscous, cold soils (Timofeeva et al., 2024). This reaction isn’t always beneficial, though. For instance, excessive or inappropriately timed microbial auxin signaling may inadvertently disrupt endogenous hormonal gradients during prolonged stress, impeding primary root extension or adversely altering lateral root architecture. Similarly, the consequences of microbially generated ABA signaling are highly dependent on the host genotype and the intensity of the environmental setting.

A distinct growth-defense trade-off is required during extended stress periods because prolonged high-level ABA accumulation can limit carbon absorption and cause growth arrest, even if accurate stomatal gating and osmotic adaptations depend on tight regulation of ABA. One important tactic for lowering substantial fitness costs in the midst of these complex interactions is the microbial control of the stress-induced ethylene spike. Acute chilling frequently causes plants to overproduce ethylene, which severely restricts root development and causes autocatalytic cellular senescence. Microbial 1-aminocyclopropane-1-carboxylate (ACC) deaminase functions as a very effective metabolic sink by cleaving the immediate ethylene precursor ACC into α-ketobutyrate and ammonia (Shahid et al., 2023). This targeted enzymatic intervention allows the host to preserve structural biomass and chlorophyll integrity without completely compromising its innate capacity to sense stress by selectively lowering the detrimental ethylene signaling pathway (Zhakypbek et al., 2026). Their adaptive success ultimately depends on the integration of these microbial interventions with endogenous salicylic acid (SA) and jasmonic acid (JA) pathways, which control defensive gene expression while avoiding the fitness trade-offs associated with unchecked stress signaling (Makris et al., 2026).

### 4.1 Redox homeostasis and oxidative quenching

Cold-induced metabolic disturbance at the host-endophyte interface causes a fast and dangerous build-up of intracellular ROS (Yang et al., 2017), mainly superoxide radicals (O_2_) and H_2_O_2_, endangering membrane fluidity and genomic DNA integrity. Psychrotolerant microbial partners reduce this risk by serving as external metabolic catalysts. The bacterial overexpression of important oxidative defense genes, such as *sodA* (superoxide dismutase) and *katG* (catalase-peroxidase), directly triggers the systemic activation of the host’s enzymatic and non-enzymatic detoxification pathways. This microbially mediated redox priming results in a transcriptomic shift in the host plant by upregulating endogenous genes encoding ascorbate peroxidase (APX1) and glutathione reductase (GR) and reprogramming baseline secondary metabolism to maintain elevated cellular concentrations of defensive phenolics and radical-scavenging flavonoids. Thus, this multi-genomic coordination preserves systemic cellular integrity and significantly lowers lipid peroxidation, as indicated by a significant reduction in malondialdehyde (MDA) buildup, by shielding the structural lipid bilayer from oxidative denaturation (González et al., 2024). The entire holobiont operates as a coordinated defensive unit, successfully preventing brief oxidative bursts from progressing into irreversible cellular damage by closely coordinating microbial gene expression with host transcriptional feedback.

### 4.2 Transcriptional reprogramming and systemic memory

The shift from passive survival to systemic adaptability is made possible by host transcriptional reprogramming at the genomic level. According to recent studies, cold-responsive microbial signaling may have an impact on the highly conserved CBF/DREB (C-repeat Binding Factor/Dehydration-Responsive Element-Binding) pathway, which functions as an essential upstream master switch for downstream COR (Cold-Responsive) genes (Fadiji et al., 2023; Hwarari et al., 2022). The degree of direct microbial causation within the core ICE1-CBF-COR cascade remains a growing topic of inquiry rather than a well-established universal mechanism; however, localized studies suggest that some psychrotolerant inoculants may alter the expression kinetics of these transcription factors. This altered expression cascade downstream stimulates the creation of protective molecular chaperones, such as late embryogenesis abundant (LEA) proteins, which aid in shielding vital cellular machinery from dehydration caused by cold (Kim et al., 2024; Storey and Storey, 2023). To better classify these systemic, non-pathogenic microbial therapeutics, the term Microbially Induced Systemic Tolerance (MIST) has been developed in recent literature (Kour et al., 2024). Rather than operating as an entirely new paradigm, MIST is a particular conceptual extension of the conventional Induced Systemic Resistance (ISR) framework. While ISR often refers to the priming of plant defenses against biotic diseases, MIST expands the definition of ISR to encompass systemic priming of physiological networks against abiotic extremes like chilling and desiccation. This idea states that microbial volatile organic compounds (mVOCs) and low-molecular-weight quorum-sensing molecules act as distal chemical signals that initiate MIST cascades throughout the plant vascular system (Kanjana et al., 2026). According to Yu et al. (2022), Siddique et al. (2024), this interkingdom communication produces a kind of transcriptional memory that prepares native plant systems for noticeably faster activation throughout ensuing, varied freeze-thaw cycles. Ultimately, even though it still needs substantial field-scale validation, this multi-layered coordination, which includes hormonal rheostats, biophysical cryoprotection, and emerging genomic remodeling, highlights the potential of engineered plant-microbe interactions to function as an adaptive system for high-altitude crop resilience.

### 4.3 Signaling and regulatory networks: the systemic communication axis

The phytobiome’s resilience to low-temperature anomalies is regulated by multilayered signaling networks that integrate instantaneous biophysical detection with systemic transcriptional, hormonal, and metabolic reprogramming. This communication axis converts a passive survival response into a highly coordinated adaptive strategy by acting as a bidirectional feedback loop rather than a unilateral host response through the transfer of inter-kingdom chemical information. The plant host’s perception of cold starts at the plasma membrane, where low-temperature-induced rigidification activates mechanosensitive ion channels, resulting in a brief, rapid influx of cytosolic calcium ions (Ca^2+^) (Yuan et al., 2018). This intracellular calcium signature is decoded by downstream calcium-dependent protein kinases (CDPKs) and calcineurin B-like (CBL) proteins, which then initiate the MAPK cascade, a crucial signaling hub for cold acclimation (Wang et al., 2026).

There is growing evidence that microbial colonization can alter the amplitude and duration of these host calcium oscillations, suggesting that rhizosphere microorganisms may function as early signal modulators rather than merely passive downstream beneficiaries of host stress responses (Negi et al., 2023). ROS function as dual-role signaling molecules in conjunction with this calcium influx under heat stress. High-altitude excesses can cause catastrophic, unregulated ROS accumulation, whereas controlled, low-level oxidative bursts are crucial secondary messengers required to sustain MAPK activation. Psychrotolerant endophytes help maintain this signaling-competent redox threshold by controlling host NADPH oxidase activity and enhancing antioxidant buffering capabilities, which preserves signaling fidelity during fluctuating daily temperature gradients (Ţocu et al., 2025).

### 4.4 Hormonal crosstalk and volatile-mediated priming

**FIGURE 3:**
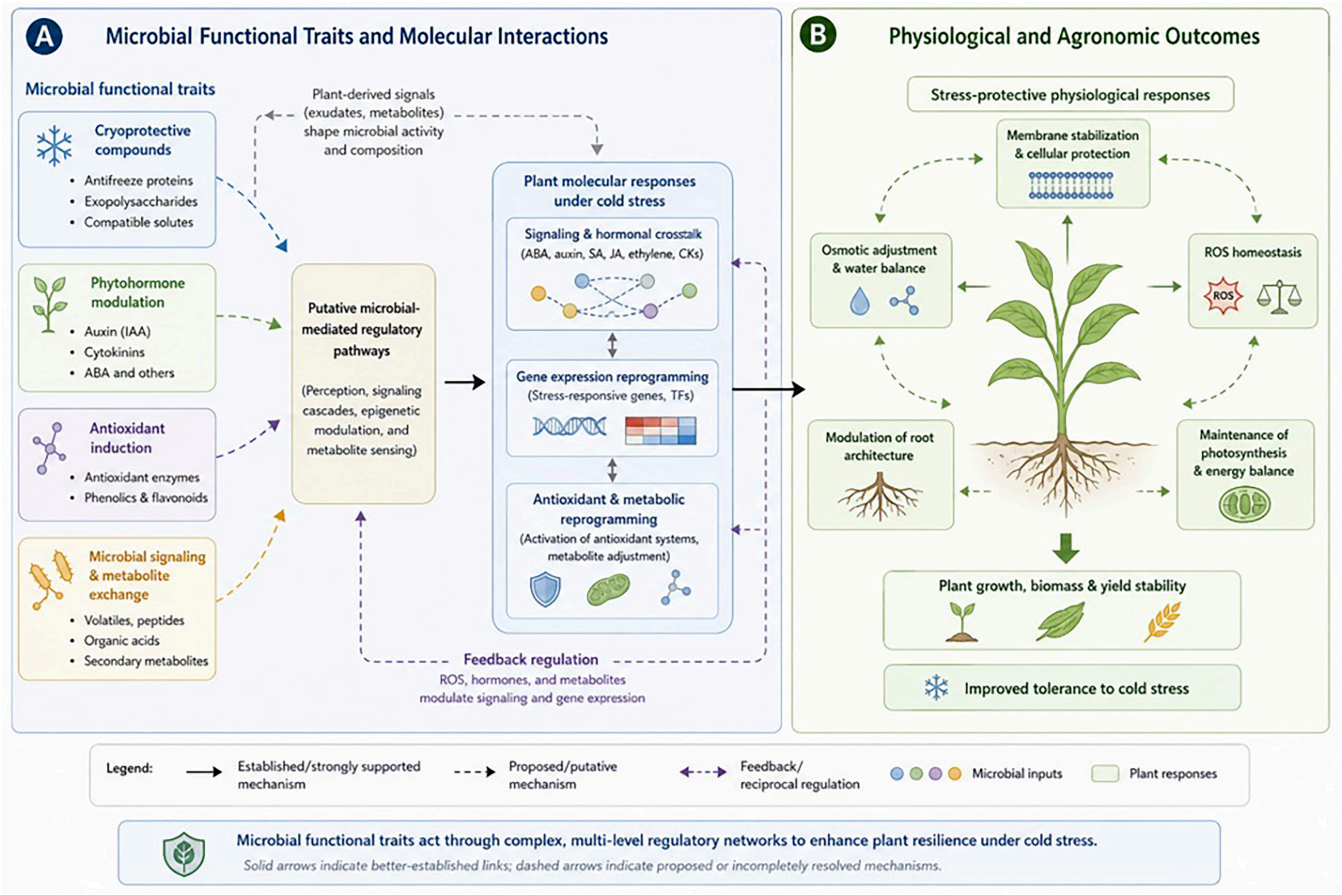
Mechanistic model of interactions between microbes and plants under cold stress. The Figure is divided into two linked modules. Major cold-adapted microbial functional traits, such as cryoprotective compounds, phytohormone modulation, antioxidant induction, and microbial metabolite exchange, are summarized in panel **(A)**. These traits may affect plant responses through potential regulatory pathways and molecular reprogramming involving signaling networks, gene expression, and metabolic modifications. Membrane stabilization, osmotic balance, reactive oxygen species (ROS) homeostasis, root architectural modification, preservation of photosynthetic function, and enhanced plant growth, biomass accumulation, and yield stability under low-temperature conditions are among the physiological and agronomic results shown in panel **(B)**. Dashed arrows highlight the complexity and feedback regulation underpinning plant-microbe interactions during cold-stress adaptation, while solid arrows show rather well-supported correlations.

This symbiotic signaling network’s physiological flexibility is further enhanced by extensive hormonal cross-talk mediated by microbial metabolites (Berrios and Rentsch, 2022). In the face of extreme climate stress, amino acid signaling-based metabolic priming is crucial for maintaining cellular homeostasis in addition to structural changes. Specifically, glutamine is an important signaling hub that optimizes carbon/nitrogen (C/N) balance through interactions with the GABA system, maintains physiological vigor during heat stress and water deprivation, and alters plant transcriptome defense genes (Tomar et al., 2026). Using synthetic consortia that can alter these host metabolic checkpoints is a precise way to lower cold-arid susceptibility. ABA is a crucial regulatory node in this network that governs stomatal dynamics and cellular osmotic equilibrium. It is believed that some microbial inoculants alter host sensitivity to endogenous ABA, maximizing the stomatal response’s quickness to abrupt temperature changes (Wei et al., 2025). The microbial production of ACC deaminase serves as a key metabolic sink that cleaves the precursor ACC to intercept the host’s stress-induced ethylene spike, hence maintaining root elongation and biomass accumulation under chilling stress (Gulati et al., 2024). In addition to direct tissue colonization, mVOCs like acetoin and 2,3-butanediol serve as long-distance aerial signals that trigger systemic changes without the need for direct physical contact. These low-molecular-weight vapors cause the expression of stress-responsive host genes by altering the JA and SA pathways to preserve photosynthetic efficiency at below-optimal temperatures (Kour et al., 2024). To achieve dependable rhizosphere buffering, bacterial N-acyl homoserine lactones (AHLs) involved in quorum sensing coordinate host defensive responses and density-dependent microbial activities on a community scale (Alum et al., 2025). Major cold-adapted microbial functional traits, such as cryoprotective compounds, phytohormone modulation, antioxidant induction, and microbial metabolite exchange, are summarized in Figure 3A. Membrane stabilization, osmotic balance, ROS homeostasis, root architectural modification, preservation of photosynthetic function, and enhanced plant growth, biomass accumulation, and yield stability under low-temperature conditions are among the physiological and agronomic results are shown in Figure 3B.

### 4.5 Transcriptional hubs and epigenetic memory

The systemic translation of these early biophysical and hormonal cues activates core plant transcriptional hubs. This genomic response depends on the ICE1-CBF-COR signaling pathway, in which the master regulator ICE1 activates C-repeat binding factors (CBFs/DREBs) to induce downstream transcription of Cold-Responsive (COR) genes required for membrane stability and cryoprotection (Jin et al., 2018). Following rhizosphere inoculation, these essential CBF and DREB transcription factors, together with auxiliary stress-responsive gene families like WRKY, NAC, and bZIP, have been associated with enhanced expression (Bharti et al., 2026). When properly integrated, these microbially driven changes contribute to the broader notion of MIST. In contrast to transient, short-term stress reactions, the current study explores the idea that MIST can use chromatin remodeling and histone modifications to build a form of temporary epigenetic memory inside the host (Siddique et al., 2024). By giving plants in unpredictable cold-arid environments greater metabolic flexibility and improved transcriptional kinetics during frequent, seasonal freeze-thaw cycles, this priming effect may maximize host fitness in unstable cryospheric ecosystems.

### 4.6 Rhizosphere mechanisms: by microbial functional traits

The stability of host plant energetics and structural development under low-temperature anomalies is largely dependent on the deployment of specific microbial functional traits and metabolic signaling hubs. The restriction of biochemical response rates in the rhizosphere is the primary physiological barrier to plant viability in cold-arid soils with kinetically limited nutrient transfer. Psychrotolerant plant growth-promoting microorganisms (PGPMs) circumvent these thermodynamic limitations by autonomous metabolic pathways that directly alter the root-soil interface. In frozen soils, specialist microbial consortia exploit low-temperature-active mineralization pathways to maintain essential macromolecular synthesis. This localized bacterial mobilization greatly increases the bioavailability of critical macronutrients and structural cofactors at the root surface, especially magnesium (Mg^2+^) and nitrogen (N).

This microbially driven food influx prevents the structural breakdown of the chlorophyll porphyrin ring and safeguards the catalytic integrity of the Rubisco enzyme complex during acute heat reductions by directly feeding the host’s primary metabolic sink downstream (Duarte et al., 2024). The maintenance of host carbon fixation rates, chlorophyll stability, and optimal Photosystem II (Fv/Fm) electron transport efficiency under cold stress can therefore be interpreted as a phenotypic reflection of the primary microbial nutrient-cycling infrastructure (Wu et al., 2018). For example, psychrotolerant isolates synthesize high-affinity catecholate and hydroxamate siderophores to sequester iron under extreme kinetic constraints under controlled conditions, while actively excreting low-molecular-weight organic acids, such as citric, oxalic, and gluconic acids, via specialized membrane transporters to chelate bound phosphate minerals (Chaudhary et al., 2022; Pradhan et al., 2025). When these lab-scale functional properties are applied to open field environments, there are significant ecological constraints. In actual alpine field conditions, this microbially driven nutrient solubilization often declines significantly due to local soil physicochemical variability (such as shifting pH and fluctuating moisture retention), intense resource competition from well-adapted native soil microbiomes (Muhammad et al., 2025), and the direct inhibitory effects of prolonged sub-zero temperatures on bacterial enzyme kinetics. Similarly, alterations in root system architecture under cryospheric stress are mostly caused by microbial signaling gradients rather than autonomous plant cell proliferation, albeit their field efficacy is still very context-specific. Chilling stress typically prevents cell elongation, leading to a catastrophic build-up of endogenous ethylene in root tissues.

Inoculation with psychrotolerant bacteria expressing ACC deaminase can offset this by generating an external metabolic sink that actively cleaves host stress-ethylene precursors (Ding et al., 2025). Concurrently, localized microbial synthesis of certain mVOCs and IAA modulates endogenous host auxin gradients. This microbially mediated hormonal reprogramming restores root plasticity and results in two significant architectural changes: increased lateral root density and faster root hair extension (Oukala et al., 2021). Instead of acting as a passive host reaction, this microbially enhanced root design physically enhances the holobiont’s absorptive surface area, enabling it to take advantage of nutrient-rich microsites and avoid resource-depleted, frozen soil zones. However, the inoculant’s ability to withstand extreme diurnal temperature swings and sustain steady colonization densities throughout the varied root-soil interface will ultimately determine the effectiveness of this architectural change in the field.

### 4.7 Hydraulic conductivity and cryo-desiccation mitigation

Cold stress often leads to a state of physiological drought (Jain et al., 2021) because frozen soil water is physically unavailable for absorption. To combat this, microbial interactions increase root hydraulic conductivity and aquaporin expression. PGPR act as a rampart against the adverse effects of drought stress (Bouremani et al., 2023). This hydraulic optimization preserves cell turgor and minimizes electrolyte leakage, a crucial marker of membrane integrity, with the help of microbial osmolyte buildup (Ren et al., 2026). Additionally, microbial EPS operates as a biophysical bridge, limiting desiccation at the root-soil contact by creating a wet microenvironment (Yadav et al., 2016). These coordinated, multi-tiered responses, which span from micro-scale molecular signaling to whole-plant biomass stability, eventually comprise the essential framework of agricultural resilience under cold-arid conditions (Cesco et al., 2026; Singh et al., 2024). Instead of acting as distinct physiological mechanisms, the combination of microbially driven metabolic priming, biophysical rhizosphere changes, and structural root modifications stabilizes host plant fitness during high cryospheric stress.

Nevertheless, the transfer of these symbiotic characteristics from controlled laboratory to open agroecosystems is still restricted by local microbial competition and environmental unpredictability. To overcome these field-scale translational challenges, cold-arid agriculture must shift its emphasis from open-ended, single-strain inoculations to the predictive engineering of core functioning microbiomes. By forming artificial consortia with overlapping functional characteristics and robust colonization kinetics, this prediction paradigm offers a clear road map for guaranteeing consistent agricultural productivity in the face of growing cryospheric instability.

## 5 Metagenomics: from taxonomic surveys to functional potential

Our understanding of the cryospheric phytobiome has undergone a paradigm shift due to the shift from conventional culture-dependent isolation to high-throughput multi-omics and enhanced culturomics. Despite historical assumptions that often limited culture-dependent techniques to capturing a small fraction of total microbial diversity, recent advances in high-throughput cultivation, microfluidics, and mimicked natural-media formulations—collectively referred to as culturomics—have significantly increased the recoverability and functional screening of previously resistant psychrophilic lineages (Nam et al., 2023). When paired with shotgun and amplicon metagenomics, these complementary toolkits go beyond basic phylogenetic cataloging (Younas et al., 2025) to map the functional potential and environmental responsiveness of the high-altitude endosphere and rhizosphere (Thingujam et al., 2025). Importantly, metagenomic mining demonstrates that low-temperature durability does not require a rigid, deterministic Minimal Functional Genome or a solid structural blueprint.

Instead, cryospheric fitness reflects a highly dynamic genomic architecture governed by transcriptome plasticity, horizontal gene transfer (HGT), and complex, overlapping regulatory networks. Studies conducted across the Tibetan and Himalayan plateaus have shown that cold-arid adaptation is driven by the enriched presence and fluid regulation of gene clusters devoted to the synthesis of cryoprotective EPS, CSPs, and ice-binding AFPs (Gupta et al., 2020). Fundamental studies on cryospheric habitability have shown that these structural and regulatory adaptations sustain microbial life across the planet’s vast cold biomes (Singh, 2021). The rapid overexpression of CSPs serves as a primary molecular switch during abrupt freeze-thaw cycles, preventing translation arrest and stabilizing mRNA structures.

Concurrently, multi-omic datasets demonstrate that specialized carbohydrate-active enzymes (CAZymes) and psychrophilic cold-active hydrolases maintain environmental flexibility. By performing high substrate turnover rates at below-freezing temperatures that would typically stop conventional kinetic routes, these microbially generated enzymes sustain organic matter decomposition and ongoing nutrient flux in frozen soil matrices (Ashcroft et al., 2025). Instead of just documenting these traits as static attributes, modern research employs these functional profiles to inform predictive rhizosphere engineering. This approach focuses on creating synthetic consortia based on strong horizontal gene transmission and dynamic functional redundancy rather than assuming fixed taxonomic or genomic inputs.

### 5.1 Cross-omics crosstalk and integration: the regulatory bridge

A comprehensive systems-biology framework is required to combine multi-omics layers in order to methodically interpret the high-altitude phytobiome. This approach is crucial for resolving the basic ecological distinctions between genomic potential (the mere presence of a functional gene inside a metagenome) and metabolic actuality (the actual in situ transcriptional, translational, and enzymatic execution of that gene under field stress). Shotgun metagenomics effectively maps the functional blueprint of the rhizosphere for specific features such as phosphate solubilization (pqqC), nitrogen fixation (nifH), and cold-shock regulation (cspA). It cannot, however, confirm whether these pathways are actively operating in the face of shifting cryospheric conditions (Nam et al., 2023; Younas et al., 2025). To bridge this gap, downstream metatranscriptomics and metaproteomics—which reflect the rhizosphere’s true dynamic response—must be superimposed.

**FIGURE 4:**
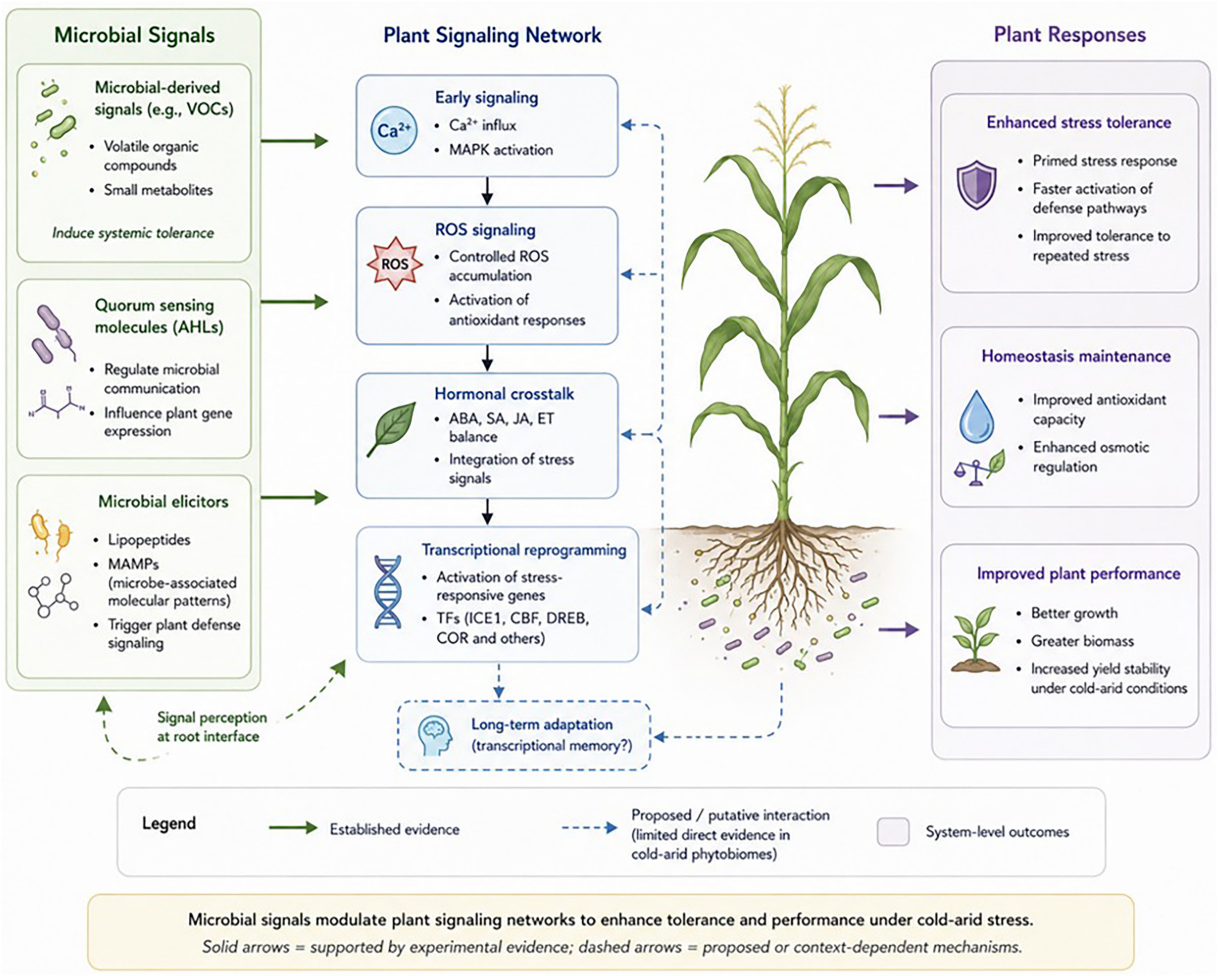
Cold-stress plant-microbe signaling, regulation networks, and adaptation. Microbial signals, including volatile organic compounds (VOCs), quorum-sensing molecules, and microbial elicitors (Zehra et al., 2021), are perceived at the root interface and influence plant signaling networks involving calcium influx, ROS signaling, hormonal crosstalk, and transcriptional reprogramming. These regulatory processes contribute to enhanced stress tolerance, maintenance of cellular homeostasis, and improved plant growth and productivity under cold-arid conditions. Solid arrows indicate interactions supported by experimental evidence, whereas dashed arrows represent proposed or incompletely resolved mechanisms requiring further validation.

Cross-omics data sets and their integration (Figure 4) show that the exact previous up-regulation of related microbial defense cassettes frequently determines the activation kinetics of host plant COR and DREB transcription factor networks (Bharti et al., 2026). When exposed to freezing temperatures, psychrotolerant endophytes rapidly up-regulate critical oxidative and osmotic protection transcripts, such as *sodA* and *gshC*, much before host plants begin initiating similar protective transcripts, according to metatranscriptomic tracking (Wani et al., 2018). This multi-genomic coordination sets off a cross-kingdom signaling cascade: the microbial transcripts generate low-molecular-weight elicitors that pierce the host cell wall, causing host MAPK cascades and altering the transcription of endogenous plant genes (Jalmi and Sinha, 2015; Wang et al., 2026).

Metabolomics captures the physical manifestation of this multi-genomic cooperation by tracking the holobiont’s final Functional Output. Metabolomic profiling provides concrete evidence of cross-kingdom interaction by identifying specific, microbially regulated chemical fluxes that directly impact physical stress tolerance. For example, the rhizosphere’s localized buildup of non-reducing disaccharides such as trehalose and certain proline isomers functions as an external osmotic buffer, stabilizing cell-surface proteins and preserving root cell turgor against desiccation (Berrios and Rentsch, 2022). In the host plant tissues, this microbial activity simultaneously causes a systemic metabolic change that propels the high-density synthesis of phenolics and flavonoids that scavenge radicals and protect the lipid bilayer from oxidative denaturation (González et al., 2024).

By structurally mapping the networks linking a microbial functional gene cluster (Metagenomics) to an active metabolic signaling flux (Metabolomics) that systematically reprograms host plant transcription (Transcriptomics), researchers can transition from open-ended, descriptive inventories to predictive, targeted intervention strategies. This integrated multi-omics roadmap provides practical support for SynComs’ logical architecture. Instead of employing arbitrary single-strain inoculants, which frequently fail because of natural soil competition, designers can produce multi-species SynComs with verified functional redundancy, overlapping transcriptional triggers, and consistent metabolic outputs (Kour et al., 2024). By ensuring regular colonization and dependable stress-buffering kinetics, this multi-layered assemblage offers a strong engineering blueprint for sustaining agricultural productivity across unstable, cold-arid ecosystems (Singh et al., 2024).

### 5.2 Meta-transcriptomics: the regulatory response of microbial symbionts

Metatranscriptomics provides the critical temporal resolution needed to differentiate between active, *in situ* functional flux and dormant metagenomic potential under freezing anomalies. Instead of a general shutdown, the transcriptional landscape of the alpine rhizosphere undergoes a highly coordinated reprogramming dominated by psychrotolerant microbial transcription kinetics following an acute temperature drop. This acute bacterial response is primarily caused by the rapid up-regulation of alternative sigma factors, including σ^B^ (encoded by *rpoS*) and σ^E^, which reroute RNA polymerase transcription away from normal growth genes and toward multi-cistronic cold-stress regulons (Rey-Campos et al., 2022). To dynamically maintain cell turgor and permeability against extracellular ice nucleation, bacterial two-component regulatory networks, such as the membrane-bound thermosensor EnvZ/OmpR cascade, undergo rapid conformational changes that initiate the transcription of genes regulating the composition of outer-membrane porins. *Bacillus* and *Pseudomonas* strains swiftly undergo vital metabolic alterations that have an immediate impact on the rhizosphere’s chemistry.

Metatranscriptomic datasets show that the acdS gene, which codes for 1-aminocyclopropane-1-carboxylate deaminase, and the pqq operon, which regulates pyrroloquinoline quinone-dependent mineral solubilization, both have rapid, low-temperature-responsive transcription (Chandwani and Amaresan, 2022). Instead of acting as a passive chemical buffer, the quick deployment of these microbial RNAs acts as a significant metabolic trigger. By drawing down local ethylene precursors and initiating the rapid extrusion of organic acids, these bacterial transcriptional changes actively modify the root-soil boundary layer. This upstream microbial transcriptomic acceleration primes the host plant’s internal signaling environment by optimizing the downstream activation kinetics of the conserved plant DREB/CBF-COR pathway and avoiding the metabolic lag that typically results in cold-shock mortality in uninoculated crops (Kim et al., 2024).

### 5.3 Proteomics: the structural basis of resilience and metabolic reprogramming of the psychrophilic proteome

While transcripts display rapid directional shifts, metaproteomics shows the true structural and functional reality of cold adaptation by quantifying the steady-state abundance of stable, catalytically active proteins. The microbial proteome has two primary physical challenges under subfreezing temperatures: the rigidification of the lipid bilayer and the thermodynamic stability of secondary structures in mRNA and proteins, which combined halt translation. The proteomes of cold-arid rhizosphere bacteria are significantly enriched in low-molecular-weight cold-shock domain proteins (CSPs, such as CspA and CspG), which function as RNA chaperones to prevent the formation of inhibitory mRNA secondary structures at freezing temperatures to prevent translation arrest (Liu et al., 2019). To enable continued ribosomal transit, cold-active ATP-dependent RNA helicases (like DeaD/CsdA) aggressively unwind cold-stabilized RNA loops. Important proteomic processes for cold adaptation include: (i) RNA stabilization: Csps and DeaD helicases prevent inhibitory mRNA secondary structures; (ii) Protein fold continuity: the multi-subunit complexes GroEL/GroES and DnaK/DnaJ prevent toxic protein misfolding and aggregation; and (iii) Membrane fluidization: Desaturases alter fatty acid profiles (C_16:0_ to C_16:1_) to prevent lipid rigidification. Maintaining macromolecular conformation requires a significant proteome investment in highly conserved chaperone networks. Psychrotolerant endophytes up-regulate the GroEL/GroES and DnaK/DnaJ tracks, two multi-subunit ATP-dependent folding complexes that actively refold cold-denatured proteins in the bacterial cytoplasm and stabilize developing polypeptides (George et al., 2024).

A basic metabolic shift toward localized, energy-efficient pathways for nutrition digesting is mechanistically consistent with this structural preservation. Quantitative proteomic profiling indicates that under cryospheric stress, microbial pathways for nitrogen absorption (via glutamate synthase and ammonium transporters) and membrane-lipid fluidization (via acyl-lipid desaturases that convert saturated fatty acids to cis-unsaturated variants) are highly prioritized. The microbial proteome’s structural and metabolic resilience directly supports the stability of the larger holobiont. These microbial proteins systematically lower systemic stress in the host tissue by maintaining continuous bacterial cell division and metabolic output at below-freezing temperatures. This leads to a parallel build-up of LEA proteins and cryoprotective dehydrins in the plant partner (Gupta et al., 2022).

### 5.4 Metabolomics: the symbiotic rhizosphere interface and metabolic flux

The metabolome captures the immediate biochemical interface of the phytobiome and represents the downstream effects of multi-genomic interactions in real time. High-resolution GC-MS and LC-MS profiling demonstrate that while microbial metabolites, particularly low-molecular-weight polyols and disaccharides like mannitol, raffinose, and trehalose, can accumulate significantly during freezing episodes to function as osmotic stabilizers and cryoprotectants, their functional efficacy is strictly limited by environmental context, concentration thresholds, and host-specific compatibility (Salam et al., 2023). Rather than being generally beneficial remedies, these exogenously produced compounds exhibit complex, dual-faceted behaviors in cold-arid rhizosphere settings. Microbially mobilized solutes, for instance, shield the host cell membrane from ice-nucleation damage at optimal concentrations. However, an excessive accumulation may inadvertently cause feedback inhibition in primary host carbon metabolism or disturb localized root-soil hydraulic conductivity gradients.

According to Li et al. (2023), the microbially generated metabolic priming is very susceptible to variations in soil moisture profiles and daily temperature swings. It is distinguished by higher rates of accumulation of specific amino acid isomers (such as proline) and radical-scavenging flavonoids. Under severe, simultaneous desiccation stress, these metabolic fluxes might shift from costly energy drains that hinder basic vegetative development to protective osmoprotectant pathways. Similar levels of phenotypic heterogeneity are shown when microbial mVOCs such acetoin and 2,3-butanediol are used. The signaling fidelity of these aerosol vapors is strongly dependent on the physical matrix of the soil, despite the fact that they are crucial long-distance cues that can modify host transcriptomes and increase systemic tolerance without requiring direct physical contact (Yang Y. et al., 2026).

In strongly compacted or wet cold-arid soils, the diffusion kinetics of these volatile signals are drastically decreased, frequently bringing their concentration below the threshold required to trigger host defense mechanisms. Due to the high genetic specificity of the host’s receptor landscape, a volatile chemical signature that successfully primes a cold-tolerant alpine landrace may not result in any physiological reaction or even localized phytotoxicity in an unadapted crop variety. Therefore, characterizing the metabolomic interface requires going beyond generic claims of benefit in order to correctly map the concentration-dependent and environment-sensitive boundaries that control symbiotic efficacy in the field.

### 5.5 Computational architectures for AI-guided SynCom design

**FIGURE 5:**
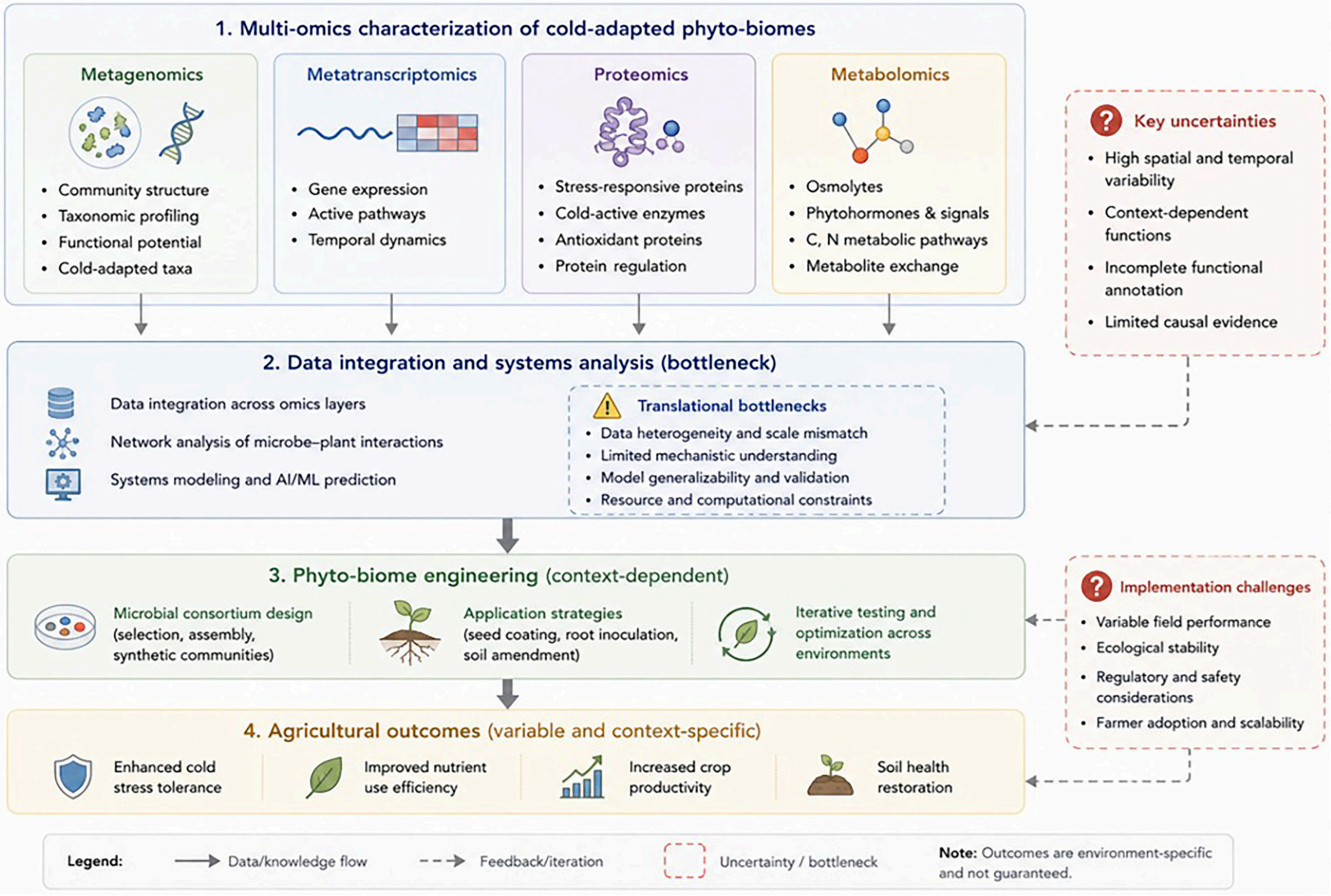
Omics-based cold-adapted phyto-biome understanding and engineering. Together, proteomics, metabolomics, metagenomics, and metatranscriptomics shed light on the composition and operation of cold-adapted plant-microbe systems. To inform phyto-biome engineering initiatives, these datasets are combined using network analysis, systems biology, and AI-assisted techniques. The framework identifies important implementation issues, translational impediments, and environmental uncertainties that affect microbial consortia development and field deployment. Enhanced cold-stress resistance, greater nutrient-use efficiency, increased productivity, and soil health restoration are examples of agricultural outcomes that are portrayed as context-dependent and necessitate iterative validation across many conditions.

By integrating multi-omics layers using systems biology, the paradigm of cryospheric phytobiome research is shifted from descriptive cataloging to a predictive and prescriptive engineering framework (Figure 5). To close the translational gap between laboratory promise and field performance, we propose a structured computational pipeline that builds robust SynComs using constraint-based Genome-Scale Metabolic Modeling (GSMM) and ML. Microbial survival, community stability, and functional output are predicted over different diurnal heat gradients using two ensemble learning models: Random Forests (RF) and Extreme Gradient Boosting (XGBoost).

These structures are parameterized using a highly specific set of ecological and physical input variables: Microbial Genomic Features: Mean kinetic temperature, diurnal temperature oscillation amplitudes (Δ T), seasonal freeze-thaw frequency data, bulk density, soil organic matter (SOM), pH, moisture retention curves (psi), and cation-exchange capacity (CEC); relative abundance vectors of functional target loci (e.g., acdS encoding ACC deaminase, the pqq operon, and fatty acid desaturases) obtained from metagenomic sequencing (Nam et al., 2023; Younas et al., 2025). Graph neural networks (GNNs), which see individual microbial taxa as nodes and metabolic exchanges as edges, are used in parallel to map the rhizosphere’s spatial and metabolic architecture.

According to Babalola et al. (2020), George et al. (2024), this approach finds specialized keystone hubs that function as essential metabolic connectors that sustain fluid nutrient exchange and whole-community network connectedness even when cold stress sharply increases community modularity. Psychrotolerant bacteria of *Arthrobacter* and *Pseudomonas* are examples of these hubs. After these hubs are mapped, FBA using multi-species GSMMs simulates the co-metabolism of the plant-microbe unit under steady-state environmental constraints at 0 °C (Passi et al., 2021). This *in silico* simulation identifies metabolic bottlenecks and resource rivalry by using specific cold-acclimation operons as molecular markers to guide the logical assembly of high-performance inoculants appropriate for certain high-altitude zones (Patyal et al., 2025; Vega-Celedón et al., 2021).

To prevent these computational models from remaining speculative, anticipated interaction topologies and cross-feeding networks must be thoroughly empirically confirmed using a two-tiered experimental workflow. First, high-throughput microfluidic droplet microhabitats are used to co-cultivate projected pairings or trios of psychrotolerant isolates under controlled heat regimes. Micro-Raman spectroscopy is used to verify calculated growth yields and track real-time metabolic exchanges. Second, validated consortia are injected into sterile, structurally similar synthetic soil microcosms that undergo simulated cryospheric freeze-thaw cycles. In order to definitively prove the mechanistic causality of specific predicted traits, target strains undergo precise CRISPR-Cas9 or Tn5 transposon mutagenesis to knock out the responsible loci (e.g., Δ acdS), confirming whether the loss of the gene disrupts the predicted network stability and host phenotypic resilience (Ding et al., 2025).

The training datasets currently available for cold-arid agroecosystems provide substantial translational obstacles, notwithstanding the potential of these digital twin frameworks. First, there are serious problems with data sparsity and geographic bias; because of their strong bias toward temperate, lowland agricultural soils, high-altitude, cryospheric ecosystems are persistently under-represented in public omics databases (Thingujam et al., 2025). An out-of-distribution generalization error occurs when conventional machine learning models are used to alpine rhizo-ecologies. Second, kinetic parameterisation gaps hinder the accuracy of FBA; the enzyme turnover rates (kcat) and substrate affinities (Km) required to constrain metabolic models are almost exclusively recorded at normal mesophilic temperatures (25 °C–37 °C). Because psychrophilic enzyme kinetics exhibit highly non-linear deceleration courses as temperatures approach freezing, using uncorrected mesophilic parameters causes serious mathematical errors in in silico calculations. It is imperative to recognize these computational limitations (Singh et al., 2024) to move AI-guided SynCom assembly from a conceptual framework into a reliable, repeatable framework for cryospheric agricultural adaptation. In response to extreme moisture deficits, plants actively seek out protective endophytes and rearrange their rhizobiome using an evolutionary cry-for-help approach (Tomar et al., 2025). The multi-omics characterization of these relationships enables the customized building of SynComs designed to optimize host metabolic reprogramming. This multi-layered approach offers a precise molecular foundation to overcome earlier lab-to-field translational challenges in stress-affected agricultural zones.

### 5.6 Technical bottlenecks and translational hurdles

Translating high-altitude phytobiome datasets into predictable agronomic outputs is still hampered by a number of interconnected technological, experimental, and ecological obstacles, despite recent computational and multi-omic advances. To ensure that laboratory-derived models successfully transition to field-scale validation in cold-arid locations, future projects must go beyond idealized workflows and systematically address these systemic limitations: biomass and extraction constraints: The essentially low microbial titers common in hyper-arid, sub-zero soils significantly hinder high-quality nucleic acid and protein isolation (Parveen et al., 2026). Low biomass yields can necessitate extensive nested PCR amplification or low-input library preparation methods, which produce large sequencing biases, over-represent dominant taxa, and artificially reduce the reported structural diversity of the rare biosphere. Furthermore, the high percentage of sand in alpine soils and the frequent presence of interfering glomalin or humic substances exacerbate extraction losses, leading to uneven cell lysis that distorts the apparent abundance of thick-walled Gram-positive psychrotolerants (Arthrobacter) relative to their gram-negative counterparts.

Culturing bottlenecks and phenotypic mismatch: Despite the rapid advancement of modern culturomics, a significant portion of basic high-altitude taxa remain unculturable *in vitro* due to a lack of understanding regarding specific cryospheric growth triggers and cross-feeding dependencies. We are still a long way from being able to physically assemble these strains into functional SynComs or structurally validate the uncultured functional potential discovered in metagenomic databases. Stochastic drift versus deterministic selection: in highly unstable, disturbed alpine agroecosystems, microbial community assembly is influenced by more than only deterministic environmental selection, such as survival of the fittest under cold stress. Instead, ecological drift—caused by random birth, death, and dispersal events—often outweighs anticipated selection pressures in patchily dispersed mountain soils. When similar crop genotypes grown in neighboring, chemically identical alpine plots form fundamentally distinct rhizosphere networks, this stochasticity causes serious repeatability problems.

Limitations of cross-study comparability: The lack of standardized sample techniques, varying sequencing depths, and highly fragmented, non-parameterized bioinformatic pipelines make it difficult to make meaningful cross-study comparisons across different mountain ranges (e.g., comparing Andean datasets with Himalayan or Tibetan plateaus) (Suyal et al., 2022).

To overcome these structural challenges, the field must prioritize the adoption of standardized bioinformatic pipelines that fully integrate environmental parameters (such as UV-B radiation indices, localized soil moisture matrices, and precise freeze-thaw frequencies). These biological and technical obstacles must be overcome in order to go beyond theoretical explanations and accomplish predictable, field-scale engineering of the cryospheric phytobiome (Figure 5).

## 6 Phytobiome engineering and agricultural applications

At the forefront of cryospheric research is the integration of scalable, field-level applications with fundamental mechanistic understanding. Phytobiome engineering, defined as the targeted, structural modification of the plant-microbe-soil interactome, offers a helpful basis for enhancing crop resilience in high-altitude cold deserts (Kushwaha et al., 2020). As climate change shortens growing seasons and increases thermal volatility in fragile mountain ecosystems, optimizing these microbial interactions is a potential means of protecting crops from acute abiotic stressors (Zeng et al., 2025). However, the application of these laboratory-designed techniques to cold-arid fields is still in its infancy since field-scale efficacy heavily depends on complicated soil matrices and unpredictable macro-environmental changes. Comparative synthesis of phytobiome engineering strategies in cold-arid agroecosystems are illustrated in Table 2.

### 6.1 Single inoculants vs. designer consortia

More and more research is being done on designed SynComs as possible replacements for traditional single-strain inoculants, which frequently cannot tolerate the climatic conditions of high-altitude environments. By integrating complementary psychrotolerant bacteria, fungi, and actinomycetes, these designer consortia aim to exploit functional redundancy to ensure that some metabolic services tolerate localized perturbations (Gurubasajar and Basaiah, 2025). Different strains within these communities offer parallel processes for nitrogen fixation, phytohormone regulation, and phosphorus solubilization. The combination of *Bacillus subtilis*, *Pseudomonas fluorescens*, and *Arthrobacter agilis* that were isolated from cold-adapted Ladakh soils is a well-known illustration of such design frameworks (Yadav et al., 2016). These strains show significant metabolic compatibility in controlled greenhouse settings, working in concert to maintain root plasticity and chlorophyll retention in local landraces of buckwheat (*Fagopyrum esculentum*) and barley (*Hordeum vulgare*) after acute, simulated frost events.

Theoretical success of such assemblies under non-equilibrium conditions has historically been associated with community robustness, the capacity of a multi-species system to maintain its core metabolic outputs through cross-feeding networks and functional overlap, even if individual members experience temporary demographic declines (Chan et al., 2017). When these well-regulated pot-culture successes are converted into scaled field applications, there are important operational and ecological challenges that are not addressed in short-term research. In closed substrates, this tripartite consortium exhibits immediate post-frost plant growth-promoting efficacy; however, virtually little longitudinal data support its long-term survival throughout actual seasonal freeze-thaw cycles in cryospheric soils. Extreme physical stress caused by high-altitude thermal volatility in open agroecosystems often results in a rapid fall in the titers of the inoculation population over succeeding winter seasons.

The wider ecological effects of introducing these high-density synthetic consortia are still poorly understood, and high-throughput high-resolution amplicon sequencing is rarely used to assess transient or permanent microbial community shifts, dysbiosis, or non-target displacements within the native rhizosphere. Without systematic monitoring of structural changes within the native soil microbiome and multi-season tracking of strain survival, such examples provide helpful proof-of-concept illustrations rather than definitive evidence of field-ready, scalable microbiome engineering (Table 2). Despite these theoretical benefits, moving from straightforward single-strain applications to sophisticated SynComs has substantial ecological risks that often impair field performance. According to Contreras-Salgado et al. (2024), a high species variety naturally raises the frequency of detrimental biotic interactions, which causes extreme ecological instability.

Exotic designer strains will face fierce microbial competition and possible niche exclusion from well-adapted local microbiomes if they are put into an open agricultural soil environment (Contreras-Salgado et al., 2024). By producing unfriendly antimicrobials or sequestering resources, native populations frequently hinder the success of designed consortia. In cold-arid soils, colonization failure is a recurring problem. When stringent environmental filtration is applied to introduced strains, less-tolerant designed species are rapidly eliminated before they form solid root connections due to abrupt decreases in soil temperature, low moisture, and severe nutrient shortages. According to Contreras-Salgado et al. (2024), the performance of these designer consortiums is very context-dependent; the key elements that determine whether a consortia cooperates or collapses include changing soil heterogeneity, different host plant genotypes, and local root exudation patterns. The functional redundancy built into SynComs in controlled settings may not produce reliable agricultural results in real-world field circumstances if these specific ecological restrictions are ignored.

### 6.2 The translational roadmap: precision delivery and endophytic priming

#### 6.2.1 Precision encapsulation and controlled-release matrices

Modern approaches use co-encapsulation of artificial consortia in biodegradable polymeric matrix, such as carboxymethyl cellulose composites and iron- or calcium-crosslinked alginate-chitosan nanogels, in place of raw liquid inoculants. These macro- and nanostructured matrices alter release kinetics from immediate, uncontrollable spikes to zero-order or multi-stage diffusion-controlled patterns. In high-altitude cold deserts, these matrices serve as physical barriers that shield encapsulated cells from hyper-arid desiccation and high-flux UV-A and UV-B radiation, preserving cell viability over protracted exposure by up to 3–4 log CFU/g compared to unformulated cells. By adjusting the cross-linking density and including stimuli-responsive functional groups (such as temperature- or pH-sensitive polymers), microbial liberation can be coordinated with specific plant growth phases or environmental cues. For instance, when soil temperatures exceed the psychrophilic metabolic threshold of 5 °C–10 °C or when root exudate concentrations (such as certain organic acids) increase, matrix breakdown and subsequent microbial release can be engineered to occur (Hu et al., 2023). This coordinated distribution enhances the efficiency of localized rhizosphere colonization by preventing early microbial washout during early-season meltwater runoff.

#### 6.2.2 Endophytic bio-priming and translational bottlenecks

An alternative method to prevent macroenvironmental changes is endophytic bio-priming, which employs natural or genetically altered cold-adapted endophytes that colonize internal host tissues. Inoculation of seeds or seedlings with psychrotolerant *Pseudomonas* and *Microbacterium* endophytes directly alters host systemic physiology, limiting lipid peroxidation and maintaining cell membrane fluidity under freezing stress by upregulating critical cold-responsive genes and transcriptional activators such as RD29A, COR15a, and C-repeat binding factors (CBFs) (Jain et al., 2021; Chaudhary et al., 2022).

Despite the potential advantage of a sheltered intra-tissue niche, endophytic engineering faces several translational obstacles that limit predicted field application:

Host compatibility and cultivar specificity: The genotype of the host significantly restricts the development of an endophytic relationship; a strain that successfully primes a lab model may be completely excluded by the strict immune responses of local landraces of buckwheat or barley, or it may be actively identified as a pathogen.

Persistence decay and in-planta competition: Newly imported endophytes must contend with an established, well-adapted native endophytic colony. Synthetic strains often fail to achieve sustained niche saturation and experience rapid population declines due to direct host vascular system antagonism or competitive exclusion.

Biosafety and regulatory restrictions: The potential for HGT of engineered traits, such as markers of antibiotic resistance or altered metabolic pathways, to spread to wild, non-target alpine flora is one of the ecological problems raised by foreign or genetically modified endophytes.

If these issues with taxonomic compatibility, persistent stability, and biological containment are not addressed, endophytic priming remains a restricted experimental strategy rather than an immediate field-ready replacement.

### 6.3 AI/ML-assisted prediction of microbial design and ecological infrastructure

Modern frameworks incorporate structured machine learning methods to analyze high-throughput multi-omics datasets (metagenomic potential paired with metabolomic flux) to move from descriptive microbiome surveys to predictive, targeted assembly. Instead of relying on broad, aspirational AI promises, predictive engineering applies a special combination of supervised and unsupervised learning techniques to analyze complex plant-microbe-soil interactomes (Tuladhar et al., 2023; Gulati et al., 2024).

First, unsupervised learning, more specifically, topological data analysis (TDA), principal coordinate analysis (PCoA), and hierarchical cluster analysis (HCA), is employed to reduce dimensionality and map the baseline structural variance of native cryospheric microbiomes across various environmental gradients. Microbial co-occurrence network analysis is then performed using specific computational frameworks such as SparCC (Sparse Correlations for Compositional Data) or SPIEC-EASI (Sparse Inverse Covariance Estimation for Ecological Association Inference). These techniques recreate dependable, non-spurious interaction patterns by eliminating compositionality artifacts, which are commonly found in amplicon sequencing datasets.

Key network topology metrics, such as high degree centrality (total direct connections), high closeness centrality (proximity to all other nodes), and low betweenness centrality (acting as a critical bottleneck/bridge), are used to computationally isolate Keystone Connector Taxa within these generative graphs. These keystone species serve as the primary metabolic hubs of the community. Once located, these hubs provide synthetic consortia with their structural framework. Supervised ensemble classifiers, such as RF, Gradient Boosted Machines (GBM), and Deep Neural Networks (DNN), are trained on labeled datasets that match microbial abundance profiles with host phenotypic outcomes (e.g., biomass increase, osmolyte accumulation) to predict the functional performance of these assemblies under particular geographic constraints, such as the hyper-arid, sub-zero conditions of Ladakh.

These models reduce the need to import non-native, environmentally inappropriate biofertilizers by predicting the agronomic efficacy of particular designer consortia combinations prior to laboratory or field validation. In order to completely advance toward the predictive engineering of climate-smart agricultural systems, contemporary frameworks increasingly rely on the convergence of digital modeling and nanotechnology. Kumar et al. (2026) claim that by integrating big data, robotics, the Internet of Things (IoT), and artificial intelligence (AI), the shift to Agriculture 5.0 maximizes agricultural production while lowering waste from synthetic inputs. By integrating smart agrochemicals, nanosensors, and machine learning models, this paradigm provides a highly adaptive mechanism for accurate pest management, real-time disease identification, and the sustainable deployment of functional bio-inputs under changing environmental conditions (Kumar et al., 2026). When applied successfully, these computationally optimized phytobiomes not only boost agricultural productivity but also actively build a strong ecological infrastructure. For example, the precise addition of keystone strains that maximize microbial EPS synthesis in fragile alpine environments physically alters the soil matrix, enhancing micro-aggregate stability and mitigating severe wind and meltwater erosion (Yadav et al., 2019). At the same time, focused microbial signaling increases root exudation, which encourages long-term carbon storage in deep soil fractions. Our combined computational and biological method offers a validated framework to stabilize crop production while actively regenerating damaged mountain soils vulnerable to desertification in high-altitude greenhouse and polyhouse environments (Afridi et al., 2022b).

### 6.4 Addressing the validation gap: biosafety, ecological ethics, and regulation

Although targeted phytobiome engineering has enormous theoretical and laboratory potential, implementing these systems in open agricultural fields requires overcoming major regulatory challenges, uneven field performance, and unresolved ecological risks. In subsequent implementations, short-term agronomic metrics must be replaced by a comprehensive validation plan based on the following pillars:

#### 6.4.1 Quantifying ecological risk and native microbiome

There is a clear risk of inadvertent microbiome disruption when high-titer synthetic consortia or modified endophytes are purposefully introduced into delicate alpine habitats. Because native cryospheric microbiomes are highly specialized and structurally balanced to endure extreme nutrition and temperature constraints, they are especially vulnerable to alien biological inputs. Introduced strains may cause severe non-target impacts such as resource monopoly, direct competitive exclusion of slow-growing native psychrophiles, or disruption of essential native co-occurrence networks. This might lead to localized dysbiosis, which would alter the soil’s ecosystem indefinitely. Therefore, long-term ecological monitoring must be incorporated into standard validation methods. High-resolution, multi-omics sequencing pipelines must be used over multiple vegetative cycles to track the spatial-temporal persistence of introduced strains, their decay rates during seasonal freeze-thaw cycles, and their direct structural effects on the alpha- and beta-diversity of the native rhizosphere.

#### 6.4.2 Horizontal gene transfer and biosafety containment

In microbiome engineering, containing altered genetic material is a major unresolved issue, particularly when employing genetically optimized or bio-primed endophytes. High cell densities in commercial inoculants or host vascular systems raise the possibility of HGT by plasmid-mediated conjugation, transduction, or natural transformation (Raghuvanshi et al., 2026). The possibility of synthetic functional traits (such as improved stress-tolerance pathways, particular metabolic loops, or selection markers like antibiotic resistance genes) escaping into the natural soil matrix or unintentionally entering native alpine flora and weed populations is very high. This could lead to ecological imbalances or unpredictable evolutionary shifts in protected mountain biomes. To counteract these containment failures, advanced biological safety measures must be developed, such as tailored synthetic auxotrophy or genomic kill-switches, that ensure the introduced strain self-destructs if it deviates from the designated host crop or rhizosphere zone.

#### 6.4.3 Regulatory frameworks, ethical Governance, and multi-stakeholder collaboration

It is necessary to develop standardized, context-specific regulatory frameworks for high-altitude agriculture in order to develop field-scale applications. Current bio-safety approval models, which are often created for temperate, low-altitude farming systems, rarely take into account the unique vulnerabilities of alpine ecosystems. There must be clear, evidence-based laws governing risk tiering, environmental release permits, and intellectual property protection for native microbial strains. According to Kumar et al. (2026), the shift from isolated laboratory discoveries to scalable, commercial smart bioformulations requires a well-aligned multi-stakeholder ecosystem. Legislators, agronomists, soil scientists, and molecular microbiologists must collaborate to create standardized testing frameworks (Patyal et al., 2025). This cooperative approach ensures that phytobiome engineering is governed by strict ecological ethics by striking a balance between the long-term biological preservation of vulnerable mountain soils and current agricultural output.

Understanding how complex substances interact with soil ecology is crucial for developing efficient delivery systems. As nano-reactors, nanoparticles (NPs) protect synthetic consortia and improve nutrient cycling. Raghuvanshi et al. (2026), however, draw attention to their dual behavior: while low doses may induce beneficial responses in agricultural microbes by boosting gene expression, initiating ROS signaling, and encouraging nitrogen fixation and metabolite production, high doses may be toxic. Therefore, incorporating nanotechnology into soil health management without disturbing the rhizosphere requires eco-friendly designs and standardized life cycle assessments.

As climate variability accelerates structural changes in cold-arid agroecosystems, phytobiome engineering (Table 2) must transition from empirical single-strain inoculations (Strategy 1) to computationally optimized, multi-omic synchronized frameworks (Strategies 6 and 8). Despite their high initial translational readiness, single strains’ susceptibility to competitive exclusion under erratic freeze-thaw cycles limits open-field efficacy (TRL 7–8). Conversely, multi-omic validated SynComs and AI-driven predictive deployments offer unparalleled mechanistic precision, but they require significant validation to bridge the translational gap from sterile lab matrices to high-altitude real-world situations.

## 7 Challenges and future perspectives

The transition from fundamental mechanistic understanding to the sustainable use of cold-arid phyto-biome technologies is currently hampered by a complicated web of administrative, ecological, and technical challenges. Even while laboratory-scale studies have established the fundamental stress-buffering abilities of psychro-tolerant bacteria, the extreme environmental instability of high-altitude deserts poses a significant obstacle to the long-term durability and replicability of these treatments. The true efficacy of engineered microbial consortia will depend on their ability to scale within the high-complexity dynamics of the cryosphere, as the majority of current data come from short greenhouse or pot studies that lack the ecological realism of open-field systems (Wendel, 2022). The ecological stability of imported strains, which must contend with well-adapted native microbiota for limited resources while withstanding the mechanical demands of frequent freeze-thaw cycles and severe desiccation, continues to be a crucial bottleneck (Muñoz-Carvajal et al., 2023). Before predictive engineering and AI-guided Synthetic Communities (SynComs) can become useful field practices, the following eight basic structural problems must be fixed:

### 7.1 Microbial instability and competitive exclusion in cold soils

When SynComs or strains that have been optimized in the lab are introduced into real-world fields, they are immediately barred from competing. Native soil communities have adapted to local temperature and nutrient limitations for thousands of years. This offers them a distinct home-court advantage over introduced bioinoculants, which frequently fail to colonize the rhizosphere at the critical population threshold (10^6^ CFU per gram of root tissue) required to produce substantial agronomic benefits.

### 7.2 Lack of standardized omics and bioinformatic pipelines

The data utilized to train predictive AI algorithms determines their quality. Currently, cold-desert multi-omics datasets are widely distributed and seldom standardized. Most public repositories lack sufficient representation of extreme psychrophilic microbiomes. An analytical bottleneck resulting from inadequate functional annotation of unique psychrophilic metabolic genes and the absence of standard protocols for obtaining low-biomass microbial DNA/RNA from frozen or extremely dry soils impedes repeatable machine-learning predictions.

### 7.3 Intense seasonal microbial turnover

Unlike stable temperate zones, cold-arid ecosystems see sharp, seasonal shifts in microbial communities. When winter freezes arrive, large portions of the vegetative active microbiome collapse, shifting the community structure to spore-forming or highly inactive states. Introduced SynComs intended for spring crop development rarely survive these challenging seasonal transition stages, requiring costly yearly re-applications rather than building long-lasting, self-sustaining multi-season networks.

### 7.4 Acute environmental stochasticity

Extreme environmental changes describe high-altitude agroecosystems. Inoculants have to deal with intense UV and solar radiation, abrupt, prolonged dry periods, and soil temperatures that can vary by more than 30 °C in a single day (Shankhwar et al., 2020). This physical stress rapidly breaks down exposed microbial cell walls and damages their DNA, which significantly lowers the lifespan of bioinoculants before the crop can build its early root system.

### 7.5 Poor landscape-scale reproducibility

The complex topography of mountain agriculture creates incredibly dispersed microclimates. A SynCom formulation that performs exceptionally well in one specific alluvial valley may completely fail on an adjacent terraced plot with different slope dynamics, solar exposure, or frost-free windows. This large regional variability makes it very difficult to develop commercial bio-formulations that are scalable and generalizable.

### 7.6 Regulatory barriers for engineered consortia

Although custom-designed SynComs and CRISPR-edited psychro-tolerant modules have great potential in lab settings, they face substantial regulatory challenges. The focus of existing bio-safety protocols is on single-strain, unaltered wild isolates. The approval process for multi-strain synthetic communities, especially those with manufactured elements, requires extensive risk assessments, which might delay field testing for years.

### 7.7 Long-term ecological risks to fragile alpine biomes

When many non-native microbial strains are introduced into fragile alpine environments, long-term ecological concerns arise. There is a significant risk of HGT between introduced inoculants and native psychrophiles. This could unintentionally disrupt the delicate local functioning ecology, change soil nitrogen cycle rates, or remove indigenous beneficial bacteria that are crucial for sustaining wild alpine flora.

### 7.8 Economic feasibility for mountain agriculture

Finally, the practical difficulties of mountain farming are a major cost barrier. Smallholder farmers sometimes face low profit margins and poorly linked supply chains in remote, hilly areas. Delivering advanced, living bioinoculants to hard-to-reach terraced fields is typically too expensive to be commercially viable without significant, long-term governmental subsidies. For storage and delivery, these bioinoculants typically need temperature-controlled cold chains.

## 8 Future strategic directions

A quick transition to novel delivery methods and precise bio-formulations is required to address these persistent problems. A practical method for preserving microbial functioning throughout seasonal changes is the creation of cryo-protective carriers and polymeric encapsulation techniques that correspond with certain soil physicochemical profiles (Madushani et al., 2026). Additionally, the creation of regional microbial biobanks is necessary to preserve native isolates that are both functionally characterized and naturally compatible with the agro-ecological circumstances of the area. The Interpretive Gap between high-throughput multi-omics data and real plant physiological responses is a major drawback. Because of the significant soil heterogeneity and fluctuating microbial activity that distinguish genotype from phenotypic expression, the current focus on descriptive metagenomics occasionally fails to prove causality (Mohamedikbal et al., 2025). To enable reliable meta-analyses and cross-study comparisons, future research directions must highlight the integration of seasonally synchronized sampling approaches with standardized bioinformatics procedures. To understand community degradation and the breakdown of beneficial metabolic networks, field-scale perturbations such as modified irrigation schedules or anthropogenic fertilizer inputs must be examined. To go beyond static data and develop a predictive understanding of community adaptation, long-term field research backed by ecological modeling and metagenome-resolved population dynamics is necessary. The advent of AI and ML pipelines that can synthesize large multi-omic datasets to create Designer Consortia suited for certain crop-climate niches will significantly enhance this progress.

The ethical and biosafety ramifications of introducing modified organisms into delicate alpine ecosystems must continue to be at the forefront of scientific discussion as research advances toward genome editing and metabolic engineering to boost microbial resistance (Ashcroft et al., 2025). Cutting-edge science, regional regulations, and local farming practices must all be closely integrated for long-term adoption. A collaborative strategy that combines conventional agricultural knowledge with cutting-edge biotechnology resources is needed to navigate the many regulatory requirements for biofertilizer certification across several countries. The next 10 years of Smart Microbiome Agriculture will ultimately be defined by the combination of molecular biology and predictive analytics, altering cold desert ecosystems through ecological restoration, food sovereignty, and microbially-driven climate resistance (Afridi et al., 2022b).

## 9 Conclusion

### 9.1 The phyto-biome as an ecological renaissance

Cold-arid phytobiome engineering is a major development in sustainable agriculture that provides a precise, predictive framework for farming in the world’s worst places, going beyond just cataloging literature. The investigation of bioinoculants other than single-strain isolates is the main originality of this study. Instead, we show how high-altitude areas like the Ladakh plateau, which act as evolutionary crucibles, are home to highly evolved, multi-kingdom microbial networks. By mapping these systems using multi-omics, functional ecology, and predictive modeling, this review provides a novel way to understand how psychro-tolerant communities collaborate to maintain nutrient flux, build soil aggregates, and program plant transcriptomes under multi-tier abiotic stress.

Translating these molecular discoveries into field-ready biotechnology is the next immediate step. The practical future of climate-smart farming depends on the creation of Designer Consortia, which are precision-managed microbial assemblies built using predictive AI and machine learning to match specific crop-climate niches. In order to address field instability and seasonal community declines, future research must focus on advanced distribution technology. We will be able to protect introduced strains from sudden freeze-thaw cycles and extreme dryness by using cryoprotective carriers, target-specific polymeric encapsulation, and the creation of local microbial biobanks, ensuring their successful colonization of the root zone. This paradigm shift toward Smart Microbiome Agriculture sees the soil as a dynamic, living infrastructure rather than an inert growth substrate.

To effectively implement these technologies, we must integrate traditional farming knowledge, supportive regional policies, and high-resolution genomic data. By assisting the invisible microbial networks that sustain life at the edge of the livable earth, phyto-biome engineering can guarantee food sovereignty and climate resilience for vulnerable mountain people. This approach produces long-term productivity by working in tandem with the natural microbial systems that have sustained the cryosphere for millennia rather than exploiting them.

## References

[B1] Abeysinghe G. De Zoysa H. K. S. Bamunuarachchige T. C. Zakeel M. C. M. (2022). “Cold-tolerant and cold-loving microorganisms and their applications,” in *Trends of Applied Microbiology for Sustainable Economy*, eds Soni R. Suyal D. C. Yadav A. N. Goel R. (Amsterdam: Elsevier), 301–335. 10.1016/B978-0-323-91595-3.00006-9

[B2] Afridi M. S. Ali S. Salam A. César Terra W. Hafeez A. Sumaira et al. (2022a). Plant microbiome engineering: hopes or hypes. *Biology* 11:1782. 10.3390/biology11121782 36552290 PMC9774975

[B3] Afridi M. S. Javed M. A. Ali S. De Medeiros F. H. V. Ali B. Salam A. et al. (2022b). New opportunities in plant microbiome engineering for increasing agricultural sustainability under stressful conditions. *Front. Plant Sci.* 13:899464. 10.3389/fpls.2022.899464 36186071 PMC9524194

[B4] Ali S. Glick B. (2024). Root exudate metabolites alter food crops microbiomes, impacting plant biocontrol and growth. *Crops* 4 43–54. 10.3390/crops4010004

[B5] Alsharif W. Saad M. M. Hirt H. (2020). Desert microbes for boosting sustainable agriculture in extreme environments. *Front. Microbiol.* 11:1666. 10.3389/fmicb.2020.01666 32793155 PMC7387410

[B6] Alum E. U. Gulumbe B. H. Izah S. C. Uti D. E. Aja P. M. Igwenyi I. O. et al. (2025). Natural product-based inhibitors of quorum sensing: a novel approach to combat antibiotic resistance. *Biochem. Biophys. Rep.* 43:102111. 10.1016/j.bbrep.2025.102111 40641742 PMC12242448

[B7] Ashcroft E. Poma M. Tischler D. Munoz-Munoz J. (2025). Mining metagenomes from extremophiles as a resource for novel glycoside hydrolases for industrial applications. *Methods Enzymol.* 714 45–60. 10.1016/bs.mie.2025.02.008 40288852

[B8] Babalola O. O. Fadiji A. E. Enagbonma B. J. Alori E. T. Ayilara M. S. Ayangbenro A. S. (2020). The nexus between plant and plant microbiome: revelation of the networking strategies. *Front. Microbiol*. 11:548037. 10.3389/fmicb.2020.548037 33013781 PMC7499240

[B9] Bao Y. Dolfing J. Guo Z. Chen R. Wu M. Li Z. et al. (2021). Important ecophysiological roles of non-dominant Actinobacteria in plant residue decomposition, especially in less fertile soils. *Microbiome* 9:84. 10.1186/s40168-021-01032-x 33827695 PMC8028251

[B10] Barros-Rodríguez A. Rangseekaew P. Lasudee K. Pathom-Aree W. Manzanera M. (2021). Impacts of agriculture on the environment and soil microbial biodiversity. *Plants* 10:2325. 10.3390/plants10112325 34834690 PMC8619008

[B11] Berrios L. Rentsch J. D. (2022). Linking reactive oxygen species (ROS) to abiotic and biotic feedbacks in plant microbiomes: the dose makes the poison. *Int. J. Mol. Sci*. 23:4402. 10.3390/ijms23084402 35457220 PMC9030523

[B12] Bharti S. Gandy J. Hengge I. B. Christofferson B. Persans M. Chakrabarti M. et al. (2026). Roles of transcription factors in mediating abiotic stress responses in cereals. *Plant Stress* 19:101160. 10.1016/j.stress.2025.101160

[B13] Bhatt R. Raghuvanshi M. Kalia R. (2016). Achieving sustainable livelihood in cold arid regions of India through multienterprise options. *Ann. Arid Zone* 54 3–4. 10.56093/aaz.v54i3-4.63044

[B14] Bhattacharyya A. Mavrodi O. Bhowmik N. et al. (2023). Bacterial biofilms as an essential component of rhizosphere plant-microbe interactions. *Methods Microbiol.* 53 3–48. 10.1016/bs.mim.2023.05.006 38415193 PMC10898258

[B15] Bouremani N. Cherif-Silini H. Silini A. et al. (2023). Plant growth-promoting rhizobacteria (PGPR): a rampart against the adverse effects of drought stress. *Water* 15:418. 10.3390/w15030418

[B16] Camaille M. Fabre N. Clément C. Ait Barka E. (2021). Advances in wheat physiology in response to drought and the role of plant growth promoting rhizobacteria to trigger drought tolerance. *Microorganisms* 9:687. 10.3390/microorganisms9040687 33810405 PMC8066330

[B17] Cesco S. Alzate Zuluaga M. Y. Cavani L. et al. (2026). Roots and the rhizosphere: a perspective on the hidden engine of regenerative, antifragile, and digitally enabled agriculture. *Farming Syst.* 4:100199. 10.1016/j.farsys.2026.100199

[B18] Chan S. H. J. Simons M. N. Maranas C. D. (2017). SteadyCom: predicting microbial abundances while ensuring community stability. *PLoS Comput. Biol.* 13:e1005539. 10.1371/journal.pcbi.1005539 28505184 PMC5448816

[B19] Chandwani S. Amaresan N. (2022). Role of ACC deaminase producing bacteria for abiotic stress management and sustainable agriculture production. *Environ. Sci. Pollut. Res. Int.* 29 22843–22859. 10.1007/s11356-022-18745-7 35050477

[B20] Chaudhary P. Agri U. Chaudhary A. Kumar A. Kumar G. (2022). Endophytes and their potential in biotic stress management and crop production. *Front. Microbiol*. 13:933017. 10.3389/fmicb.2022.933017 36325026 PMC9618965

[B21] Chauhan M. Pandey A. (2024). Microbial diversity in cold desert ecosystem: a review and bibliometric analysis. *AAZ* 63 1–12. 10.56093/aaz.v63i3.152428

[B22] Cha-um S. Rai V. Takabe T. (2019). “Proline, glycinebetaine, and trehalose uptake and inter-organ transport in plants under stress,” in *Osmoprotectant-Mediated Abiotic Stress Tolerance in Plants*, eds Hossain M. Kumar V. Burritt D. Fujita M. Mäkelä P. (Cham: Springer), 201–223.

[B23] Colin C. Akpo E. Ait-Ameur L. et al. (2025). Encapsulation of biocontrol agents in alginate hydrogels: a strategy for enhanced stability and release. *Carbohydrate Polym. Technol. Appl.* 12:101036. 10.1016/j.carpta.2025.101036

[B24] Contreras-Salgado E. A. Sánchez-Morán A. G. Rodríguez-Preciado S. Y. Díaz-Zaragoza M. (2024). Multifaceted applications of synthetic microbial communities: advances in biomedicine, bioremediation, and industry. *Microbiol. Res.* 15 1709–1727. 10.3390/microbiolres15030113

[B25] Costa O. Y. A. Raaijmakers J. M. Kuramae E. E. (2018). Microbial extracellular polymeric substances: ecological function and impact on soil aggregation. *Front. Microbiol.* 9:1636. 10.3389/fmicb.2018.01636 30083145 PMC6064872

[B26] Crivelli X. B. Cundon C. Bonino M. P. Sanin M. S. Bentancor A. (2024). The complex and changing genus bacillus: a diverse bacterial powerhouse for many applications. *Bacteria* 3 256–270. 10.3390/bacteria3030017

[B27] Deng F. Xie H. Zheng T. Yang Y. Bao X. He H. et al. (2024). Dynamic responses of soil microbial communities to seasonal freeze-thaw cycles in a temperate agroecosystem. *Sci. Total Environ*. 950:175228. 10.1016/j.scitotenv.2024.175228 39102954

[B28] Dhakar K. Pandey A. (2020). Microbial ecology from the himalayan cryosphere perspective. *Microorganisms* 8:257. 10.3390/microorganisms8020257 32075196 PMC7074745

[B29] Ding Y. Yao J. Li F. Hou L. Cheng J. Zhang R. et al. (2025). Unlocking rhizosphere phosphorus: root exudate-microbe synergy drives phosphorus activation in mixed Chinese fir species plantation. *Indust. Crops Prod.* 237:122220. 10.1016/j.indcrop.2025.122220

[B30] Duarte B. Carreiras J. A. Cruz-Silva A. Naranjo E. M. Pajuelo E. Mesa-Marín J. et al. (2024). Marine plant growth promoting bacteria (PGPB) inoculation technology: testing the effectiveness of different application methods to improve tomato plants tolerance against acute heat wave stress. *Plant Stress* 11:100434. 10.1016/j.stress.2024.100434

[B31] Ejaz M. R. Badr K. Hassan Z. U. Al-Thani R. Jaoua S. (2024). Metagenomic approaches and opportunities in arid soil research. *Sci. Total Environ*. 953:176173. 10.1016/j.scitotenv.2024.176173 39260494

[B32] Fadiji A. E. Adeniji A. Lanrewaju A. A. Adedayo A. A. Chukwuneme C. F. Nwachukwu B. C. et al. (2025). Key challenges in plant microbiome research in the next decade. *Microorganisms* 13:2546. 10.3390/microorganisms13112546 41304231 PMC12654229

[B33] Fadiji A. E. Yadav A. N. Santoyo G. Babalola O. O. (2023). Understanding the plant-microbe interactions in environments exposed to abiotic stresses: an overview. *Microbiol. Res.* 271:127368. 10.1016/j.micres.2023.127368 36965460

[B34] Fondi M. Bosi E. Giudice A. L. Fani R. (2016). “A systems biology view on bacterial response to temperature shift,” in *Grand Challenges in Biology and Biotechnology*, ed. Pabulo H. (Cham: Springer International Publishing), 523–542. 10.1007/978-3-319-13521-2_21

[B35] Geng Y. Xie F. Liu Y. Zhang S. Hu B. Wang J. et al. (2026). Multi-omics analysis of microbial succession and metabolite dynamics in cold-stored walnut kernels. *Int. J. Food Microbiol*. 447:111529. 10.1016/j.ijfoodmicro.2025.111529 41365138

[B36] George T. S. Bulgarelli D. Carminati A. Chen Y. Jones D. Kuzyakov Y. et al. (2024). Bottom-up perspective - The role of roots and rhizosphere in climate change adaptation and mitigation in agroecosystems. *Plant Soil* 500 297–323. 10.1007/s11104-024-06626-6 40969406 PMC12441093

[B37] Gharib G. Saeidiharzand S. Sadaghiani A. K. Koşar A. (2022). Antifreeze proteins: a tale of evolution from origin to energy applications. *Front. Bioeng. Biotechnol*. 9:770588. 10.3389/fbioe.2021.770588 35186912 PMC8851421

[B38] Gibbons S. M. Gilbert J. A. (2015). Microbial diversity–exploration of natural ecosystems and microbiomes. *Curr. Opin. Genet. Dev*. 35 66–72. 10.1016/j.gde.2015.10.003 26598941 PMC4852739

[B39] González M. C. Roitsch T. Pandey C. (2024). Antioxidant responses and redox regulation within plant-beneficial microbe interaction. *Antioxidants* 13:1553. 10.3390/antiox13121553 39765881 PMC11673414

[B40] Gulati A. Thakur R. Vyas P. Sharma A. Dhar H. Pal M. et al. (2024). Fostering climate-resilient agriculture with ACC-deaminase producing rhizobacterial biostimulants from the cold deserts of the Indian Himalayas. *J. Environ. Manage.* 371:123075. 10.1016/j.jenvman.2024.123075 39471599

[B41] Gupta A. Mishra R. Rai S. Bano A. Pathak N. Fujita M. et al. (2022). Mechanistic insights of plant growth promoting bacteria mediated drought and salt stress tolerance in plants for sustainable agriculture. *Int. J. Mol. Sci.* 23:3741. 10.3390/ijms23073741 35409104 PMC8998651

[B42] Gupta S. Kataki S. Chatterjee S. Prasad R. K. Datta S. Sharma S. et al. (2020). Cold adaptation in bacteria with special focus on cellulase production and its potential application. *J. Clean. Product.* 258:120351. 10.1016/j.jclepro.2020.120351

[B43] Gurubasajar N. Basaiah T. (2025). Integrating microbial consortia into biofertilizers for sustainable agriculture: enhancing plant productivity and soil health. *Arch. Agric. Environ. Sci.* 10 157–163. 10.26832/24566632.2025.1001023

[B44] Handoko R. N. S. Lin S.-Y. (2025). Integrating plant growth regulators and biostimulants to enhance resilient and sustainable raspberry and blackberry production. *Sci. Horticult.* 350:114296. 10.1016/j.scienta.2025.114296

[B45] Heredia-Ponce Z. de Vicente A. Cazorla F. M. Gutiérrez-Barranquero J. A. (2021). Beyond the wall: exopolysaccharides in the biofilm lifestyle of pathogenic and beneficial plant-associated Pseudomonas. *Microorganisms* 9:445. 10.3390/microorganisms9020445 33670010 PMC7926942

[B46] Hu M. Hei R. Guo D. Luo J. Lu C. Ma Y. et al. (2023). Shelf-life enhancement of bio-inoculants through synergistic effects of encapsulation technology and osmotic protectants. *J. Environ. Chem. Eng.* 11:110996. 10.1016/j.jece.2023.110996

[B47] Hu Y. Wang Z. Jiang H. Chen Y. Liu Q. (2022). Current knowledge and future prospects regarding microorganisms in mountain glacier ecosystems. *Adv. Earth Sci.* 37 655–670. 10.11867/j.issn.1001-8166.2022.058

[B48] Huang H. Wang Q. Yang Y. Zhong W. He F. Li J. (2024). The mycobiome as integral part of the gut microbiome: crucial role of symbiotic fungi in health and disease. *Gut Microbes* 16:2440111. 10.1080/19490976.2024.2440111 39676474 PMC11651280

[B49] Hwarari D. Guan Y. Ahmad B. Movahedi A. Min T. Hao Z. et al. (2022). ICE-CBF-COR signaling cascade and its regulation in plants responding to cold stress. *Int. J. Mol. Sci*. 23:1549. 10.3390/ijms23031549 35163471 PMC8835792

[B50] Jain R. Bhardwaj P. Pandey S. S. Kumar S. (2021). *Arnebia euchroma*, a plant species of cold desert in the himalayas, harbors beneficial cultivable endophytes in roots and leaves. *Front. Microbiol*. 12:696667. 10.3389/fmicb.2021.696667 34335527 PMC8322769

[B51] Jalmi S. K. Sinha A. K. (2015). ROS mediated MAPK signaling in abiotic and biotic stress- striking similarities and differences. *Front. Plant Sci.* 6:769. 10.3389/fpls.2015.00769 26442079 PMC4585162

[B52] Jan D. Wani U. Andrabi K. John R. (2018). Cold stress modulates osmolytes and antioxidant system in *Calendula officinalis*. *Acta Physiol. Plant.* 40 1–16. 10.1007/s11738-018-2649-0

[B53] Ji M. Kong W. Jia H. Delgado-Baquerizo M. Zhou T. Liu X. et al. (2022). Polar soils exhibit distinct patterns in microbial diversity and dominant phylotypes. *Soil Biol. Biochem.* 166:108550. 10.1016/j.soilbio.2022.108550

[B54] Jin Y. Zhai S. Wang W. Ding X. Guo Z. Bai L. et al. (2018). Identification of genes from the ICE-CBF-COR pathway under cold stress in Aegilops- Triticum composite group and the evolution analysis with those from Triticeae. *Physiol. Mol. Biol. Plants* 24 211–229. 10.1007/s12298-017-0495-y 29515316 PMC5834981

[B55] Kanjana N. Guo P. Deng Z. Ahmed M. A. Shah I. Zhang L. et al. (2026). Microbial volatile organic compounds reshape plant hormonal networks and root herbivore defense. *Curr. Plant Biol.* 45:100584. 10.1016/j.cpb.2026.100584

[B56] Khanum S. Al Tawaha A. R. Al Tawaha A. R. et al. (2023). “Arbuscular mycorrhizal fungi in alleviation of cold stress in plants,” in *Advancing Frontiers in Mycology & Mycotechnology*, eds Satyanarayana T. Deshmukh S. Deshpande M. (Singapore: Springer), 197–212.

[B57] Kim J. S. Kidokoro S. Yamaguchi-Shinozaki K. Shinozaki K. (2024). Regulatory networks in plant responses to drought and cold stress. *Plant Physiol*. 195 170–189. 10.1093/plphys/kiae105 38514098 PMC11060690

[B58] Kou X. Cao X. Wang Y. Sun Z. Ji J. Duan G. et al. (2025). Multi-source ecological water replenishment reshapes microbial community assembly and network stability in a water-scarce river. *Water Res. X* 29:100441. 10.1016/j.wroa.2025.100441

[B59] Kour D. Negi R. Khan S. S. Kumar S. Kaur S. Kaur T. et al. (2024). Microbes mediated induced systemic response in plants: a review. *Plant Stress* 11:100334. 10.1016/j.stress.2023.100334

[B60] Kumar A. Sangwan P. Kumar V. Pandey A. K. Kumar A. Chauhan P. et al. (2025). Physio-biochemical insights of endophytic microbial community for crop stress resilience: an updated overview. *J. Plant Growth Regul.* 44 2641–2664. 10.1007/s00344-024-11596-1

[B61] Kumar C. Singh A. K. Tomar A. Amami R. Ghazouani H. (2026). “Artificial intelligence in sustainable and smart agrochemicals,” in *Artificial Intelligence in Chemical Engineering*, ed. F. Sher (Amsterdam: Elsevier), 443–469. 10.1016/B978-0-443-34076-5.00023-7

[B62] Kumar N. Kumar A. Jeena N. et al. (2020). “Factors influencing soil ecosystem and agricultural productivity at higher altitudes,” in *Microbiological Advancements for Higher Altitude Agro-Ecosystems & Sustainability*, eds Goel R. Soni R. Suyal D. C. (Singapore: Springer), 55–70.

[B63] Kushwaha P. Kashyap P. Kuppusamy P. (2020). “Microbes for cold stress resistance in plants: mechanism, opportunities, and challenges,” in *Microbiological Advancements for Higher Altitude Agro-Ecosystems & Sustainability. Rhizosphere Biology*, eds Goel R. Soni R. Suyal D. (Singapore: Springer), 269–292.

[B64] La Rosa R. Molin S. Johansen H. K. (2025). *Pseudomonas aeruginosa*: persistence beyond antibiotic resistance. *Trends Microbiol.* 33, 1076–1084. 10.1016/j.tim.2025.05.004 40441928

[B65] Lauritano C. Rizzo C. Lo Giudice A. Saggiomo M. (2020). Physiological and molecular responses to main environmental stressors of microalgae and bacteria in polar marine environments. *Microorganisms* 8:1957. 10.3390/microorganisms8121957 33317109 PMC7764121

[B66] Lee S. Y. Cho K. S. (2025). Enhancement of the phytoremediation performance in heavy metal-contaminated soil using a multifunctional EPS-producing bacterium Kosakonia sp, W18. *Environ. Res.* 274:121355. 10.1016/j.envres.2025.121355 40064344

[B67] Leung P. M. Bay S. K. Meier D. V. Chiri E. Cowan D. A. Gillor O. et al. (2020). Energetic basis of microbial growth and persistence in desert ecosystems. *mSystems* 5:e00495-19. 10.1128/mSystems.00495-19 PMC7159902 32291352

[B68] Li X. B. Hu C. M. Li C. H. Ji G. Y. Luo S. Z. Cao Y. et al. (2023). LC/MS- and GC/MS-based metabolomic profiling to determine changes in flavor quality and bioactive components of *Phlebopus portentosus* under low-temperature storage. *Front. Nutr*. 10:1168025. 10.3389/fnut.2023.1168025 37457983 PMC10349180

[B69] Liu X.-Y. Sun D.-W. Tian Y. (2025). Revolutionizing cryopreservation: bio-based cryoprotectants, mechanisms, and advanced applications for sustainable pan-food systems. *Trends Food Sci. Technol.* 163:105164. 10.1016/j.tifs.2025.105164

[B70] Liu Y. Lu S. Liu K. Wang S. Huang L. Guo L. (2019). Proteomics: a powerful tool to study plant responses to biotic stress. *Plant Methods* 15:135. 10.1186/s13007-019-0515-8 31832077 PMC6859632

[B71] Madushani G. R. D. S. Wu X. Jayasinghe W. H. Wang Q. Vinit K. Hao G. F. (2026). Harnessing eCISs for precision phytomicrobiome engineering and biocontrol. *FEMS Microbiol. Rev.* 50:fuag006. 10.1093/femsre/fuag006 41758122 PMC12961389

[B72] Makris A. Daloglu B. Gutjahr C. Ried-Lasi M. K. (2026). Abscisic acid at the crossroad of abiotic stress responses and plant-microbe interactions. *PLoS Pathog*. 22:e1013872. 10.1371/journal.ppat.1013872 41575940 PMC12829796

[B73] Mehta D. Vyas S. (2023). Comparative bio-accumulation of osmoprotectants in saline stress tolerating plants: a review. *Plant Stress* 9:100177. 10.1016/j.stress.2023.100177

[B74] Messer L. F. Lee C. E. Wattiez R. Matallana-Surget S. (2024). Novel functional insights into the microbiome inhabiting marine plastic debris: critical considerations to counteract the challenges of thin biofilms using multi-omics and comparative metaproteomics. *Microbiome* 12:36. 10.1186/s40168-024-01751-x 38389111 PMC10882806

[B75] Mohamedikbal S. Al-Mamun H. A. Bestry M. S. Batley J. Edwards D. (2025). Integrating multi-omics and machine learning for disease resistance prediction in legumes. *Theor. Appl. Genet.* 138:163. 10.1007/s00122-025-04948-2 40579624 PMC12204941

[B76] Montejano-Ramírez V. Ávila-Oviedo J. L. Campos-Mendoza F. J. Valencia-Cantero E. (2024). Microbial volatile organic compounds: insights into plant defense. *Plants* 13:2013. 10.3390/plants13152013 39124131 PMC11314544

[B77] Muhammad M. Wahab A. Waheed A. Hakeem K. R. Mohamed H. I. Basit A. et al. (2025). Navigating climate change: exploring the dynamics between plant-soil microbiomes and their impact on plant growth and productivity. *Glob. Chang Biol.* 31:e70057. 10.1111/gcb.70057 39924996

[B78] Mukhia S. Kumar A. Kumari P. Kumar R. (2022). Psychrotrophic plant beneficial bacteria from the glacial ecosystem of Sikkim Himalaya: genomic evidence for the cold adaptation and plant growth promotion. *Microbiol. Res*. 260:127049. 10.1016/j.micres.2022.127049 35504236

[B79] Munir N. Hanif M. Abideen Z. El-Keblawy A. Radicetti E. Mancinelli R. et al. (2022). Mechanisms and strategies of plant microbiome interactions to mitigate abiotic stresses. *Agronomy* 12:2069. 10.3390/agronomy12092069

[B80] Muñoz-Carvajal E. Araya-Angel J. P. Garrido-Sáez N. González M. Stoll A. (2023). Challenges for plant growth promoting microorganism transfer from science to industry: a case study from chile. *Microorganisms* 11:1061. 10.3390/microorganisms11041061 37110484 PMC10140820

[B81] Nam N. N. Do H. D. K. Loan Trinh K. T. Lee N. Y. (2023). Metagenomics: an effective approach for exploring microbial diversity and functions. *Foods* 12:2140. 10.3390/foods12112140 37297385 PMC10252221

[B82] Negi N. P. Prakash G. Narwal P. Panwar R. Kumar D. Chaudhry B. et al. (2023). The calcium connection: exploring the intricacies of calcium signaling in plant-microbe interactions. *Front. Plant Sci*. 14:1248648. 10.3389/fpls.2023.1248648 37849843 PMC10578444

[B83] Nie W. He Q. Guo H. Zhang W. Ma L. Li J. et al. (2024). Arbuscular mycorrhizal fungi: boosting crop resilience to environmental stresses. *Microorganisms* 12:2448. 10.3390/microorganisms12122448 39770651 PMC11677594

[B84] Oukala N. Aissat K. Pastor V. (2021). Bacterial endophytes: the hidden actor in plant immune responses against biotic stress. *Plants* 10:1012. 10.3390/plants10051012 34069509 PMC8161118

[B85] Panwar A. Ghosh K. Reddy L. J. Pandey S. John J. (2025). “Phytobiome modulation to control plant diseases,” in *Phytomicrobiome and Stress Regulation*, eds Ilyas N. Khan A. Sayyed R. Z. Mix K. D. (Amsterdam: Elsevier), 449–465. 10.1016/B978-0-443-33594-5.00006-8

[B86] Parveen S. Shafi Z. Shahid M. Iqbal M. Z. Naznine F. Ansari M. I. (2026). Omics-driven insights into soil microbial diversity and phytopathogen interactions for sustainable agriculture and food security. *J. Basic Microbiol*. 66:e70155. 10.1002/jobm.70155 41693402

[B87] Passi A. Tibocha-Bonilla J. D. Kumar M. Tec-Campos D. Zengler K. Zuniga C. (2021). Genome-scale metabolic modeling enables in-depth understanding of big data. *Metabolites* 12:14. 10.3390/metabo12010014 35050136 PMC8778254

[B88] Patyal U. Bala R. Kaur M. Faizan M. Alam P. (2025). Phyto-microbiome engineering: designing plant-microbe interactions for improved crop performance. *Microbe* 6:100272. 10.1016/j.microb.2025.100272

[B89] Pradhan N. Singh S. Saxena G. Pradhan N. Koul M. Kharkwal A. C. et al. (2025). A review on microbe-mineral transformations and their impact on plant growth. *Front. Microbiol*. 16:1549022. 10.3389/fmicb.2025.1549022 40822401 PMC12350396

[B90] Prakash S. Verma S. Kumari M. Bhattacharya N. (2026). The hydro-microbial nexus and ecological stratification in himalayan agroecosystems. *Proc. Natl. Acad. Sci.* 10.1007/s40011-026-01772-6

[B91] Qian Z. He L. Li F. (2024). Understanding cold stress response mechanisms in plants: an overview. *Front. Plant Sci.* 15:1443317. 10.3389/fpls.2024.1443317 39568458 PMC11576170

[B92] Raghuvanshi N. Kumar C. Parmar K. Tomar A. Kumar V. (2026). The nano-reactor and the microbial enzyme activation: unmasking the dualistic power of nanoparticles for sustainable soil health. *J. Soil Sci. Plant Nutr.* 10.1007/s42729-026-03240-6

[B93] Rajguru B. Shri M. Bhatt V. D. (2024). Exploring microbial diversity in the rhizosphere: a comprehensive review of metagenomic approaches and their applications. *3 Biotech* 14:224. 10.1007/s13205-024-04065-9 PMC11379838 39247454

[B94] Ren N. Liu J. Wang H. Liu Z Liu X Li G. et al. (2026). Combined transcriptomic and proteomic analysis reveals the response mechanisms of alfalfa to freezing stress. *Front. Plant Sci.* 16:1682825. 10.3389/fpls.2025.1682825 41658559 PMC12872475

[B95] Rey-Campos M. Ríos-Castro R. Gallardo-Escárate C. Novoa B. Figueras A. (2022). Exploring the potential of metatranscriptomics to describe microbial communities and their effects in molluscs. *Int. J. Mol. Sci.* 23:16029. 10.3390/ijms232416029 36555669 PMC9784687

[B96] Rondón J. J. Ball M. M. Castro L. T. Yarzábal L. A. (2019). Eurypsychrophilic *Pseudomonas* spp. isolated from Venezuelan tropical glaciers as promoters of wheat growth and biocontrol agents of plant pathogens at low temperatures. *Environ. Sustain.* 2 265–275. 10.1007/s42398-019-00072-2

[B97] Roychowdhury R. Das S. P. Sarkar P. Khan Z. Kumar A. Sarker U. et al. (2025). Physiological, biochemical and molecular signaling basis of cold stress tolerance in plants. *Front. Plant Sci*. 16:1707204. 10.3389/fpls.2025.1707204 41487347 PMC12757799

[B98] Salam U. Ullah S. Tang Z. H. Elateeq A. A. Khan Y. Khan J. et al. (2023). Plant metabolomics: an overview of the role of primary and secondary metabolites against different environmental stress factors. *Life* 13:706. 10.3390/life13030706 36983860 PMC10051737

[B99] Santoyo G. Guzmán-Guzmán P. Parra-Cota F. I. Santos-Villalobos S. (2021). Plant growth stimulation by microbial consortia. *Agronomy* 11:219. 10.3390/agronomy11020219

[B100] Satyakam, Zinta G. Singh R. K. Kumar R. (2022). Cold adaptation strategies in plants-An emerging role of epigenetics and antifreeze proteins to engineer cold resilient plants. *Front. Genet.* 13:909007. 10.3389/fgene.2022.909007 36092945 PMC9459425

[B101] Shaffique S. Khan M. A. Imran M. Kang S. M. Park Y. S. Wani S. H. et al. (2022). Research progress in the field of microbial mitigation of drought stress in plants. *Front. Plant Sci.* 13:870626. 10.3389/fpls.2022.870626 35665140 PMC9161204

[B102] Shahid M. Singh U. B. Khan M. S. Singh P. Kumar R. Singh R. N. et al. (2023). Bacterial ACC deaminase: insights into enzymology, biochemistry, genetics, and potential role in amelioration of environmental stress in crop plants. *Front. Microbiol*. 14:1132770. 10.3389/fmicb.2023.1132770 37180266 PMC10174264

[B103] Shankhwar A. K. Tamta P. Paliwal R. Srivastava R. K. (2020). “High altitude agro-ecosystems: challenges and opportunities,” in *Microbiological Advancements for Higher Altitude Agro-Ecosystems & Sustainability*, eds Goel R. Soni R. Suyal D. C. (Singapore: Springer), 1–13.

[B104] Siddique A. B. Parveen S. Rahman M. Z. Rahman J. (2024). Revisiting plant stress memory: mechanisms and contribution to stress adaptation. *Physiol. Mol. Biol. Plants* 30 349–367. 10.1007/s12298-024-01422-z 38623161 PMC11016036

[B105] Singh D. P. Maurya S. Satnami L. Prabha R. Sarma B. K. Rai N. et al. (2024). Roots of resistance: unraveling microbiome-driven plant immunity. *Plant Stress* 14:100661. 10.1016/j.stress.2024.100661

[B106] Singh V. (2021). “Microbial genes responsible for cold adaptation,” in *Survival Strategies in Cold-Adapted Microorganisms*, eds Goel R. Soni R. Suyal D. C. Khan M. (Singapore: Springer Nature), 101–123. 10.1007/978-981-16-2625-8_7

[B107] Smith D. D. N. Subasinghe R. M. Kehoe C. Grégoire D. S. (2026). Multi-omics provides functional insights and underscores practical challenges in assessing the composition and performance of a nitrifying microbial consortium. *Appl. Environ. Microbiol*. 92:e0198425. 10.1128/aem.01984-25 41457319 PMC12838200

[B108] Storey J. M. Storey K. B. (2023). Chaperone proteins: universal roles in surviving environmental stress. *Cell Stress Chaperones* 28 455–466. 10.1007/s12192-022-01312-x 36441380 PMC10469148

[B109] Suyal D. C. Joshi D. Kumar S. Bhatt P. Narayan A. Giri K. et al. (2022). Himalayan microbiomes for agro-environmental sustainability: current perspectives and future challenges. *Microb. Ecol.* 84 643–675. 10.1007/s00248-021-01849-x 34647148

[B110] Thingujam D. Gouli S. Cooray S. P. Chandran K. B. Givens S. B. Gandhimeyyan R. V. et al. (2025). Climate-resilient crops: integrating AI, multi-omics, and advanced phenotyping to address global agricultural and societal challenges. *Plants* 14:2699. 10.3390/plants14172699 40941864 PMC12429994

[B111] Timofeeva A. M. Galyamova M. R. Sedykh S. E. (2024). How Do plant growth-promoting bacteria use plant hormones to regulate stress reactions? *Plants* 13:2371. 10.3390/plants13172371 39273855 PMC11397614

[B112] Tomar A. Kumar C. Parmar K. Khan N. Singh R. Dwivedi S. K. et al. (2025). Innovative metabolic reprogramming in rice: unlocking drought resilience through microbial consortia interaction and sustainable agriculture. *3 Biotech* 15:343. 10.1007/s13205-025-04513-0 PMC12433425 40955358

[B113] Tomar A. Kumar C. Parmar K. Khan N. Singh R. Dwivedi S. K. et al. (2026). Glutamine-driven nitrogen regulation and defense mechanisms in rice: insights into signaling and stress adaptation. *J. Plant Growth Regul.* 45 1736–1756. 10.1007/s00344-026-12068-4

[B114] Tuladhar S. Hussain A. Baig S. Ali A. Soheb M. Angchuk T. et al. (2023). Climate change, water and agriculture linkages in the upper Indus basin: a field study from Gilgit-Baltistan and Leh-Ladakh. *Front. Sustain. Food Syst.* 6:1012363. 10.3389/fsufs.2022.1012363

[B115] Ullah F. Ali S. Siraj M. Akhtar M. S. Zaman W. (2025). Plant microbiomes alleviate abiotic stress-associated damage in crops and enhance climate-resilient agriculture. *Plants* 14:1890. 10.3390/plants14121890 40573878 PMC12196735

[B116] Vaghela N. R. Jotaniya V. B. Gohel S. D. (2026). A review on enhancing crop resilience in adverse environmental conditions: unveiling the action between plant growth-promoting rhizobacteria and crops. *Microbe* 10:100651. 10.1016/j.microb.2025.100651

[B117] Varadharajan V. Rajendran R. Muthuramalingam P. Runthala A. Madhesh V. Swaminathan G. et al. (2025). Multi-Omics approaches against abiotic and biotic stress-a review. *Plants* 14:865. 10.3390/plants14060865 40265800 PMC11944711

[B118] Vega-Celedón P. Bravo G. Velásquez A. Cid F. P. Valenzuela M. Ramírez I. et al. (2021). Microbial diversity of psychrotolerant bacteria isolated from wild flora of andes mountains and patagonia of chile towards the selection of plant growth-promoting bacterial consortia to alleviate cold stress in plants. *Microorganisms* 9:538. 10.3390/microorganisms9030538 33807836 PMC7998784

[B119] Wang R. Meng J. Yang S. Sun B. Qian W. He Y. (2026). Calcium-dependent protein kinases in plants: structure, signaling, and multifaceted regulatory roles in development and stress adaptation. *Int. J. Mol. Sci*. 27:1843. 10.3390/ijms27041843 41751983 PMC12941087

[B120] Wani M. Jan D. Qazi H. John R. (2018). Cold stress induces biochemical changes, fatty acid profile, antioxidant system and gene expression in *Capsella bursa* pastoris L. *Acta Physiol. Plant* 40:167. 10.1007/s11738-018-2747-z

[B121] Wei Y.-S. Javed T. Liu T.-T. Ali A. Gao S. (2025). Mechanisms of abscisic acid (ABA)-mediated plant defense responses: an updated review. *Plant Stress* 15:100724. 10.1016/j.stress.2024.100724

[B122] Wendel U. (2022). Assessing viability and stress tolerance of probiotics-a review. *Front. Microbiol*. 12:818468. 10.3389/fmicb.2021.818468 35154042 PMC8829321

[B123] Wu J. Nadeem M. Galagedara L. Thomas R. Cheema M. (2022). Recent insights into cell responses to cold stress in plants: signaling, defence, and potential functions of phosphatidic acid. *Environ. Exp. Bot.* 203:105068. 10.1016/j.envexpbot.2022.105068

[B124] Wu Y. J. Ren C. Tian Y. Liu P. Bai Y. J. Lai Z. R. et al. (2018). Photosynthetic gas-exchange and PSII photochemical acclimation to drought in a native and non-native xerophytic species (*Artemisia ordosica* and *Salix psammophila*). *Ecol. Indicat.* 94 130–138. 10.1016/j.ecolind.2018.06.040

[B125] Yadav A. N. Kour D. Sharma S. Sachan S. G. Singh B. Saxena A. K. et al. (2019). *Psychrotrophic Microbes: Biodiversity, Mechanisms of Adaptation, and Biotechnological Implications in Alleviation of Cold Stress in Plants.* Cham: Springer, 219–253.

[B126] Yadav A. N. Sachan S. G. Verma P. Saxena A. K. (2016). Bioprospecting of plant growth promoting psychrotrophic Bacilli from the cold desert of north western Indian Himalayas. *Indian J. Exp. Biol.* 54 142–150. 26934782

[B127] Yang C.-X. Chen S.-J. Hong X.-Y. Wang Z. Wu H. Yang Y. et al. (2025). Plant exudates-driven microbiome recruitment and assembly facilitates plant health management. *FEMS Microbiol. Rev.* 49:fuaf008. 10.1093/femsre/fuaf008 40158196 PMC12007450

[B128] Yang D. Y. Li M. Ma N. N. Yang X. H. Meng Q. W. (2017). Tomato SlGGP-LIKE gene participates in plant responses to chilling stress and pathogenic infection. *Plant Physiol. Biochem*. 112 218–226. 10.1016/j.plaphy.2017.01.006 28092850

[B129] Yang J. Tang M. Zhao H. (2026). Physiological mechanisms of plant growth-promoting rhizobacteria in enhancing abiotic stress tolerance of vegetable crops: a review. *Plants* 15:686. 10.3390/plants15050686 41829718 PMC12987201

[B130] Yang Y. Xu C. Lin D. Zheng C. Dai X. Wang N. et al. (2026). Acetoin and 2,3-butanediol differentially restructure fungal and bacterial communities and their links to host transcription in the rhizosphere of a medicinal plant. *Biology* 15:403. 10.3390/biology15050403 41823831 PMC12984207

[B131] Yarzábal L. A. (2014). “Cold-tolerant phosphate-solubilizing microorganisms and agriculture development in mountainous regions of the world,” in *Phosphate Solubilizing Microorganisms: Principles and Application of Microphos Technology*, eds Khan M. S. Zaidi A. Musarrat J. (Cham: Springer International Publishing), 113–135.

[B132] Younas M. U. Zuo S. Qasim M. Ahmad I. Feng Z. Malik T. et al. (2025). Multi-omics approaches in plant biology: decoding agronomic traits for sustainable agriculture. *Plant Stress* 18:101118. 10.1016/j.stress.2025.101118

[B133] Yu Y. Gui Y. Li Z. Jiang C. Guo J. Niu D. (2022). Induced systemic resistance for improving plant immunity by beneficial microbes. *Plants* 11:386. 10.3390/plants11030386 35161366 PMC8839143

[B134] Yuan P. Yang T. Poovaiah B. W. (2018). Calcium signaling-mediated plant response to cold stress. *Int. J. Mol. Sci.* 19:3896. 10.3390/ijms19123896 30563125 PMC6320992

[B135] Zehra A. Raytekar N. A. Meena M. Swapnil P. (2021). Efficiency of microbial bio-agents as elicitors in plant defense mechanism under biotic stress: a review. *Curr. Res. Microb. Sci.* 2:100054. 10.1016/j.crmicr.2021.100054 34841345 PMC8610294

[B136] Zeng Q. Hu H. W. Ge A. H. Xiong C. Zhai C. C. Duan G. L. et al. (2025). Plant-microbiome interactions and their impacts on plant adaptation to climate change. *J. Integr. Plant Biol*. 67 826–844. 10.1111/jipb.13863 39981843

[B137] Zhakypbek Y. Kossalbayev B. D. Tursbekov S. Tursbekova G. Berdaliyeva Z. Belkozhayev A. M. (2026). Application of beneficial bacteria to enhance plant drought resilience. *Plants* 15:753. 10.3390/plants15050753 41829784 PMC12987184

[B138] Zhang X. Yang Z. Wang L. Yue Y. Wang L. Yang X. et al. (2023). The effects of plant growth-promoting rhizobacteria on plants under temperature stress: a meta-analysis. *Rhizosphere* 28:100788. 10.1016/j.rhisph.2023.100788

[B139] Zhao M. Wang L. Wang J. Jin J. Zhang N. Lei L. et al. (2020). Induction of priming by cold stress via inducible volatile cues in neighboring tea plants. *J. Integr. Plant Biol*. 62 1461–1468. 10.1111/jipb.12937 32275096

[B140] Ţocu G. Ştefãnescu B. I. Stavăr Matei L. Ţocu L. (2025). Phagocyte NADPH oxidase NOX2-derived reactive oxygen species in antimicrobial defense: mechanisms, regulation, and therapeutic potential-a narrative review. *Antioxidants* 15:55. 10.3390/antiox15010055 41596113 PMC12837977

