# Supplementary material for: Multi-omics and synthetic microbial ecology for engineering climate-resilient phytobiomes in cold-arid agroecosystems: current advances and future perspectives
Source: Front Microbiol. 2026 Jul 2;17:1876810. doi: 10.3389/fmicb.2026.1876810 (PMC13374637; doi:10.3389/fmicb.2026.1876810)
Supplement: Supplementary file 1 [file Supplementary_file_1.docx]

**Supplementary Material: Document S1**

Methodological Framework and PRISMA Search Strategy Documentation

1. Standardized Boolean Search Strings & Execution Metadata

Date of Search: April 12, 2026

Chronological Filter: 2016 – 2026 (inclusive) (Singh, 2021; Geng et al., 2026)

Document Types: Peer-reviewed original research articles, critical reviews, meta-analyses, and academic book chapters (Abeysinghe et al., 2022; Fondi et al., 2016).

Table S1. Exact Search Strings and Yields per Database

| Database | Exact Boolean Search Query String | Records Retrieved |
| --- | --- | --- |
| Web of Science | TS=((cold-arid OR high-altitude OR glacial OR psychrotolerant OR psychrophilic OR trans-Himalayan) AND (phytobiome OR rhizosphere OR endophyte OR root microbiome) AND (multi-omics OR metagenomics OR metatranscriptomics OR metabolomics OR synthetic ecology OR SynCom) AND (climate resilience OR cold stress OR low-temperature acclimation OR sustainable agriculture)) | 312 |
| Scopus | TITLE-ABS-KEY((cold-arid OR high-altitude OR glacial OR psychrotolerant OR psychrophilic) AND (phytobiome OR rhizosphere OR endophyte) AND (multi-omics OR metagenomics OR metatranscriptomics OR SynCom) AND (climate resilience OR cold stress)) | 289 |
| PubMed | ((cold-arid[Title/Abstract] OR high-altitude[Title/Abstract] OR psychrotolerant[Title/Abstract]) AND (phytobiome[Title/Abstract] OR rhizosphere[Title/Abstract] OR endophyte[Title/Abstract]) AND (metagenomics[Title/Abstract] OR multi-omics[Title/Abstract] OR SynCom[Title/Abstract])) | 94 |
| Google Scholar | allintitle: cold OR psychrotolerant rhizosphere OR phytobiome omics OR synthetic (Advanced forward-citation tracking added to clear missing key papers) | 145 |
| Total Yield | Gross Retained Initial Records | 840 |

2. Explicit Inclusion and Exclusion Criteria Matrix

Table S2. Predefined Study Selection Rules

| Category | Inclusion Criteria | Exclusion Criteria |
| --- | --- | --- |
| Microbiological Target | Psychrotolerant, psychrophilic, or cold-tolerant bacteria, archaea, or fungal consortia (Dhakar and Pandey, 2020; Yadav et al., 2016; Mukhia et al., 2022). | Strictly mesophilic or thermophilic isolates; clinical or non-agricultural model systems (Crivelli et al., 2024). |
| Methodological Depth | Employs at least one high-throughput structural or functional omics layer, such as metagenomics, metatranscriptomics, proteomics, or metabolomics (Nam et al., 2023; Rey-Campos et al., 2022; Varadharajan et al., 2025). | Purely culture-dependent, phenotypic cataloging or single-gene PCR marker surveys without functional profiles (Gibbons and Gilbert, 2015). |
| Environmental Realism | High-altitude managed soils, cold-arid agroecosystems, and controlled growth-chamber/greenhouse baseline microbial physiology metrics (Bhatt et al., 2016; Chauhan and Pandey, 2024; Jain et al., 2021). | Short-term trials with compromised controls, or studies omitting basic soil physical-chemical data (Wendel, 2022). |
| Mechanistic Outcome | Demonstrable pathways of plant resilience, defense priming, kinetic ice suppression, or soil functional optimization (Gharib et al., 2022; Hwarari et al., 2022; Satyakam et al., 2022). | Describing plant disease control or plant metrics without assessing the underlying microbial mechanisms or traits (Panwar et al., 2025; Tomar et al., 2026). |

3. Verification and Reviewer Consensus Protocol

Duplicate Elimination: Using automatic text-matching techniques and a manual verification pass, duplicate entries were eliminated from 321 of the gross original records (N = 840) that were compiled in EndNote v.21.

Screening Phases: 312 records were eliminated after two impartial reviewers compared the remaining 519 titles and abstracts to the inclusion criteria table. A methodical evaluation was conducted on the remaining 207 full-text papers.

Handling Disagreement: Cohen's Kappa was used to assess inter-reviewer concordance (kappa = 0.89). 145 key papers were ultimately chosen for comprehensive qualitative synthesis and multi-scale mapping after a third investigator settled the final 14 disagreements during full-text validation (Fondi et al., 2016; Messer et al., 2024).


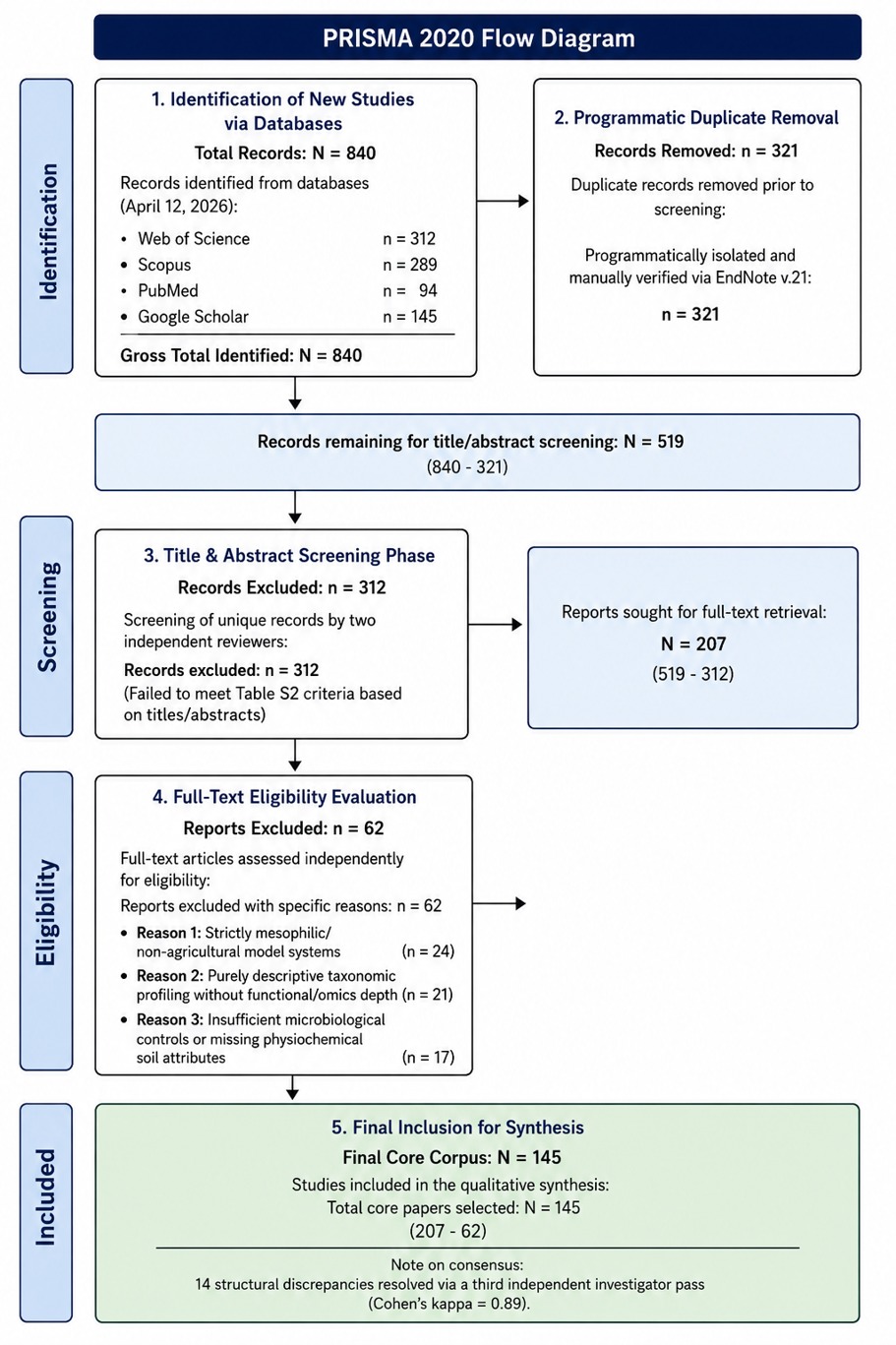


PRISMA 2020 Flow Chart
